# Reduction of late stillbirth with the introduction of fetal movement information and guidelines – a clinical quality improvement

**DOI:** 10.1186/1471-2393-9-32

**Published:** 2009-07-22

**Authors:** Julie Victoria Holm Tveit, Eli Saastad, Babill Stray-Pedersen, Per E Børdahl, Vicki Flenady, Ruth Fretts, J Frederik Frøen

**Affiliations:** 1 Division of Obstetrics and Gynecology, and Centre for Perinatal Research, Rikshospitalet University Hospital, University of Oslo, Medical Faculty, Norway; 2 Norwegian Institute of Public Health, Division of Epidemiology, Oslo, Norway; 3 Akershus University College, and University of Oslo, Medical faculty, Norway; 4 Department of Obstetrics and Gynecology, Haukeland University Hospital, Bergen, Norway; 5 Institute for Clinical Medicine, Section for Gynaecology and Obstetrics, University of Bergen, Norway; 6 Department of Obstetrics and Gynecology, University of Queensland, Mater Mothers' Hospital, South Brisbane, Australia; 7 Brigham and Women's Hospital, Div. of Maternal-Fetal Medicine, Harvard Medical School, Boston, MA, USA

## Abstract

**Background:**

Women experiencing decreased fetal movements (DFM) are at increased risk of adverse outcomes, including stillbirth. Fourteen delivery units in Norway registered all cases of DFM in a population-based quality assessment. We found that information to women and management of DFM varied significantly between hospitals. We intended to examine two cohorts of women with DFM before and during two consensus-based interventions aiming to improve care through: 1) written information to women about fetal activity and DFM, including an invitation to monitor fetal movements, 2) guidelines for management of DFM for health-care professionals.

**Methods:**

All singleton third trimester pregnancies presenting with a perception of DFM were registered, and outcomes collected independently at all 14 hospitals. The quality assessment period included April 2005 through October 2005, and the two interventions were implemented from November 2005 through March 2007. The baseline versus intervention cohorts included: 19,407 versus 46,143 births and 1215 versus 3038 women with DFM, respectively.

**Results:**

Reports of DFM did not increase during the intervention. The stillbirth rate among women with DFM fell during the intervention: 4.2% vs. 2.4%, (OR 0.51 95% CI 0.32–0.81), and 3.0/1000 versus 2.0/1000 in the overall study population (OR 0.67 95% CI 0.48–0.93). There was no increase in the rates of preterm births, fetal growth restriction, transfers to neonatal care or severe neonatal depression among women with DFM during the intervention. The use of ultrasound in management increased, while additional follow up visits and admissions for induction were reduced.

**Conclusion:**

Improved management of DFM and uniform information to women is associated with fewer stillbirths.

## Background

Maternal perception of fetal movements (FM) is a universally implemented self-screening, administered and interpreted individually by all pregnant women, with or without guidance from health care professionals [1]. Maternal reporting of decreased fetal movements (DFM) is a frequent reason for unplanned health consultations through the third trimester ranging between 4%–16% in various populations [1-3] and 5% in a previous report [2]. Pregnancies affected by DFM are at increased risk of adverse outcome such as fetal growth restriction (FGR), preterm birth and fetal death [4-9].

There is no universally accepted methodology for assessing DFM. Every method has its limitations and a "gold standard" is difficult to define. Maternal perception of FM arises first and foremost as a result of pressure against body-wall structures, and thus the mother's perception reflects gross FM or limb movements [10,11]. The proportion of movements perceived by the mother and documented during ultrasound monitoring at the same time ranges from 37% to 88% [12]. A common factor in these studies is that the mother is lying down and focusing on fetal activity. This is the only situation in which we know that maternal perception and objective measures of FM are strongly correlated with objective measures of fetal activity. Outside such a setting, both the actual frequency of movements as well as the mother's ability to perceive them are influenced by factors such as maternal position [13], activity and exercise [14], anxiety [15], stress [16], blood sugar [17], smoking [18], placenta localization [10], and obesity [19]. Parity has not been found to affect maternal perception of FM in the third trimester [10], but multiparous might be able to perceive FM earlier in pregnancy than primiparous [20]. There are significant diurnal variations in normal fetal activity, which changes gradually with gestation [10,20].

Among the attempts to define DFM, a variety of methods of FM counting with different alarm limits have been published [1,6,7]. Among these, the rule of "ten movements within 2 hours" [21]. This is the only definition of DFM based on focused maternal counting which has been both developed and tested as a screening tool in a total population, and currently the definition of DFM recommended by the American Academy of Pediatrics and the American College of Obstetricians and Gynecologists if maternal movement counting is performed [22]. Other definitions of DFM have mostly been based on counting through both rest and activity and have little evidence in support of their association with actual fetal activity. The most important clinical understanding of DFM is still the mother's own perception of a decrease [1,23-26].

There are no universally accepted guidelines for the management of DFM [7,12]. Although several studies have presented guidelines including non-stress test (NST), ultrasound examination and Doppler [2,3,5,7,22,27], most of these recommendations are based on limited evidence, as we have reviewed elsewhere [7,12].

We intended to examine two cohorts of women with DFM before and during a quality improvement intervention by implementing guidelines for management of DFM and uniform information on fetal activity to women.

## Methods

**Figure 1:**
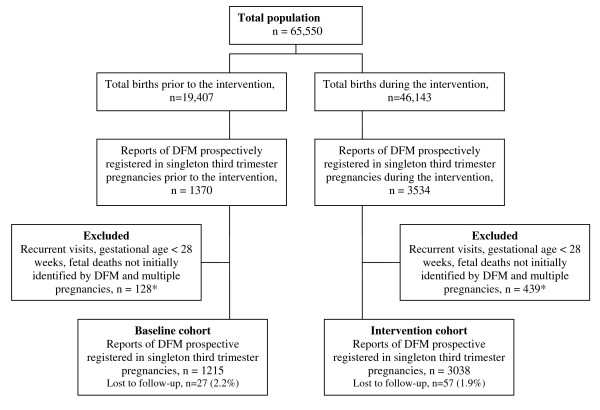
**Trial profile**. Trial profile of total births and reports of decreased fetal movements before and during the intervention *All deaths, irrespective of how they were initially identified, were included in analyses of mortality in the total population.

Women with singleton pregnancies of at least 28 weeks gestation or more who reported a concern for DFM (either by spontaneous reporting or upon questioning), were registered prospectively for quality-assurance purposes at 14 delivery units in eastern Norway and the city of Bergen. The registrations were a part of the international collaboration, Fetal Movement Intervention Assessment (Femina) [2]. Recurrent visits for DFM in already registered pregnancies were excluded as we intended to report the number of women newly reporting DFM. Data from women with a stillborn infant were obtained separately, to ensure completeness of mortality data, but stillbirths not initially identified by DFM were subsequently excluded, as were pregnancies with a gestational age under 28 weeks and multiple pregnancies (figure 1). To ensure unbiased registrations for quality-assurance of clinical practice at the individual hospital, maternal consent was not sought. The study was approved by The Regional Committees for Medical Research Ethics and Personal Data Act and advised by The Norwegian Data Inspectorate.

### Data collection

The registration period included 7 months of baseline observation followed by 17 months of intervention: from April 1, 2005 to March 31, 2007. In Norway, almost all pregnant women attend the antenatal program which is free of charge and covered by the public health-care services. The Norwegian antenatal care program is following contemporary guidelines composed by the National Institute of Clinical Excellence [28]. The community midwives and general practitioners are in charge of the antenatal program, and without the possibility to perform an NST or ultrasound examinations locally, they usually refer the concerned mothers to the nearby hospital with a maternity ward. Hence, the pregnant women in Norway typically contact maternity wards directly with any acute concerns for DFM. There are no private delivery wards in Norway. Women fulfilling the inclusion criteria were registered prospectively by the caregiver at the time the woman presented to the hospital. Pregnancy outcome were collected independently after delivery from the medical files by study coordinator at each hospital. Data were anonymized and submitted to the study-coordinating centre. DFM was defined as any woman presenting with concerns for DFM, irrespective of whether this was based on her subjective opinion or it emerged during an antenatal visit for other reasons. In addition to the registrations by our study protocol, the numbers of births and stillbirths from our population were obtained from the Medical Birth Registry in Norway to assess overall trends in stillbirth, for the most updated period available: April 2005 to December 2006.

### Guideline development

Our observations of pregnancies with DFM prior to the intervention identified significant differences in management between hospitals – none had provided the women with written information – and there were indications of co-variation between management and pregnancy outcomes [2]. Almost all hospitals would perform an NST, about half performed an ultrasound scanning, and some carried out an umbilical artery Doppler examinations [2]. The risk of adverse outcomes increased with the severity (perceived absence of DFM) and duration of DFM. Undesirable behavior was frequent, with one-third of the women did not present before an absence of FM was perceived: one-quarter of these women waited for more that 24 hours [2]. An initial survey of all 55 birth clinics in Norway found a wide range of definitions of DFM used to inform women, varying from three kicks per hour to an absence of more than 24 hours [29]. Among the fourteen participating clinics, the women received a wide range of advice in terms of normal frequency of FM: varying from 25 kicks per hour to 3 kicks per 24 hours [30].

With this in mind, a systematic review of all currently published literature was undertaken to determine the optimal management for women with DFM. A group of experts together with Chairs of midwifery and obstetrics of all participating hospitals developed a best- practice- and consensus-based approach to the best-practice management of DFM and the information provided to pregnant women. In our own quality assessment of care prior to the intervention, NST and ultrasound examination were found to be the most useful tools for fetal surveillance in DFM, while an umbilical artery Doppler examination failed to add significant information among 3014 cases of DFM. Ultrasound scanning was, by comparison, the most important tool, being the source of information in 86.2% of cases where abnormalities were detected [12]. In brief, our implemented guidelines recommended: a standard clinical evaluation for all women reporting DFM, an NST, and an ultrasound scan to quantify FM, amniotic fluid volume, and fetal anatomy and growth. A mother presenting with a concern of DFM was to be examined within two 2 hours if absence of FM was suspected, otherwise within 12 hours (guidelines published in detail) [12].

### Information for women

We developed a brochure of information that aimed to increase maternal awareness and vigilance to significant decreases in fetal activity, and to aid health-promoting behavior. This was provided as a part of the routine information given to women at the standard ultrasound assessment at 17–19 weeks of pregnancy (to which 98% of the population adhere). In addition to Norwegian, the brochure was available in Somali, Urdu, English, Turkish, and Arabic. The brochure included certain "rules of thumb" about fetal activity (additional files 1, 2, 3, 4, 5 and 6). The primary indicator of DFM was defined as her perception of a major and lasting reduction in the normal activity of her baby. In some situations the woman was advised to contact health-professionals for further examinations: 1) never to wait to the next day if the baby did not kick one day or, 2) if the baby kicked less and less in the course of a day/days, or 3) if she felt less than ten FM in 2 hours at a time of the day when the baby was usually active, and she perceived this as a reduction. As a guide to help the women to identify DFM, an invitation to use a kick chart was included. The informational brochure on FM for the mothers and new guidelines for health-care professionals were implemented in November 2005 in all hospitals included in the Femina trial.

### End points

The main outcome measures were all antepartum, intrapartum and neonatal death in the delivery room (i.e., the death occurred immediately after completion of delivery) from 28 completed weeks of gestation in women who were previously registered as having one or more episodes of DFM. As there was only one neonatal death, all deaths are called "stillbirths" in the following. The number of births and third trimester stillbirths (singleton and multiples) in the Norwegian population from the years 1999–2004 ranges between 56,374 to 59,927 births, and 2.9/1000 to 3.9/1000 stillbirths, respectively. However, as an additional 0.2/1000 to 0.4/1000 of stillbirths during the same period are registered as of unknown gestational age, this may be underestimates [31]. Secondary outcomes for women with DFM were: severe neonatal depression, defined as Apgar score of < 3 at 5 minutes postpartum; symptoms of multisystem organ failure and pH < 7 in the umbilical artery or fetal capillary scalp, if obtained; preterm birth (28° – 36^6 ^weeks); FGR (< 10th percentile of birthweight adjusted for gender and mother's height, weight, parity, and ethnicity) [32]; fetal heart rate tracings judged clinically as nonreassuring and leading to intervention in labor; oligohydramnios defined as an amniotic fluid index of < 5 cm or at < 2.5th percentile; polyhydramnios defined as an amniotic fluid index of > 25 cm or at > 97.5th percentile; investigations undertaken for reduced FM; and examinations of DFM resulting in immediate admission for induction of labor or caesarean section. Outcomes related to maternal behavior were: the number of women waiting more than 24 hours with an absence of FM or more than 48 hours with a decrease of FM before contacting health-care professionals.

### Statistical analysis

All statistical analysis were performed with SPSS version 15.0. (SPSS Chicago, IL, USA) using cross tabulations, with χ^2 ^tests and logistic regressions to find crude (unadjusted) and adjusted odds ratios (OR) with 95% confidence intervals (CI). The level of statistical significance was set at *p *< 0.05. In the multivariate analysis, all outcomes were adjusted for potential confounding factors – such as maternal age, body mass index (BMI), smoking habits, parity, and ethnicity – due to prior knowledge of their impact on pregnancy outcomes and health-promoting behavior.

## Results

Number of cases included in the baseline and intervention cohorts are described in figure 1.

The number of women presenting with DFM remained unchanged during intervention at 6.3% versus 6.6% (OR 1.05; 95% CI 0.98–1.12, *p *= 0.19), respectively. The rate of unplanned repeat visits for DFM was consistently very low, but increased from 0.3% to 0.5%, *p *= 0.002.

**Figure 2:**
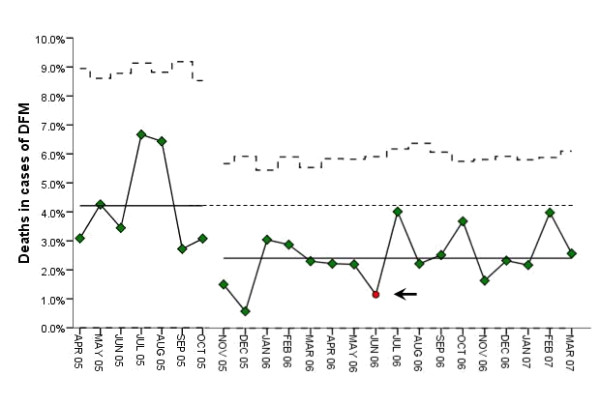
**Stillbirth rates in pregnancies presenting decreased fetal movements**. Statistical process control chart presenting the monthly stillbirth rates and means during the baseline quality assessment period and the intervention period. The arrow indicates the time (seventh month of intervention) at which a significant change was documented during the intervention.

The stillbirth rates among women with DFM were reduced by almost 50% (OR 0.51; 95% CI 0.32–0.81, *p *= 0.004)) from 4.2% (n = 50) to 2.4% (n = 73) during the intervention. Stillbirth rates among women in the entire cohort were reduced by one third from 3.0/1000 to 2.0/1000 (OR 0.67; 95% CI 0.48–0.93, *p *= 0.02). Independent data from the Medical Birth Registry in Norway, confirmed that the stillbirth rate in our total cohort of births was comparable to the rest of Norway in the baseline observation (OR 1.06; 95% CI 0.70–1.65, *p *= 0.73), and significantly lower during the intervention period (OR 0.64; 95% CI 0.47–0.87, *p *= 0.005). The intervention was followed prospectively with statistical process control charts which indicated a significant change in mortality after 7 months of intervention (arrow in figure 2), and no month during the intervention with a mortality exceeding the pre-intervention mean (figure 2). There was no increase in secondary outcomes such as preterm births, FGR, severe neonatal depression or transfers to neonatal care among women with DFM during the intervention period (table 1).

**Table 1:** Outcomes of the quality improvement intervention, N = 4253

			Univariate*			Multivariate*		
	**Baseline % (n)**	**Intervention % (n)**	**Crude OR**	**95% CI**	**P Value**	**Adjusted OR†**	**95% CI**	**P Value**
**MATERNAL BEHAVIOR IN DFM**								
**Consultation rate of DFM**	6.3 (1215)	6.6 (3038)	1.05	0.98–1.12	0.19	Not available		
**Time to contact > 24 hours in absent fetal movements**	24 (99)	18 (201)	0.70	0.53–0.92	0.01	0.73	0.53–1.00	0.05
**Time to contact ≥ 48 hours in DFM**	54 (415)	49 (897)	0.83	0.70–0.98	0.03	0.73	0.60–0.90	0.002
**EXAMINATIONS AT CONSULTATION FOR DFM**								
**Used CTG**	96 (1155)	98 (2929)	1.67	1.16–2.41	0.006	1.46	0.92–2.30	0.11
**Used ultrasound**	86 (1040)	94 (2764)	2.50	2.02–3.12	< 0.001	2.64	2.02–3.45	< 0.001
**Used Doppler**	44 (532)	47 (1415)	1.15	1.00–1.30	0.04	1.12	0.96–1.33	0.20
**CONSEQUENCES OF THE EXAMINATION FOR DFM**								
**No follow up**	63 (716)	69 (1980)	1.34	1.16–1.55	< 0.001	1.36	1.14–1.61	< 0.001
**Admissions**	14 (158)	11 (300)	0.73	0.59–0.90	0.003	0.71	0.55–0.91	0.006
**Admissions for induction**	7.0 (80)	4.9 (141)	0.69	0.52–0.92	0.01	0.68	0–49–0.96	0.03
**Admissions for emergency section**	1.8 (21)	1.2 (35)	0.66	0.38–1.14	0.14	0.73	0.40–1.59	0.43
**PREGNANCY OUTCOMES**								
**Non-reassuring heart rate tracings in labor (DFM)**	11 (130)	14 (398)	1.27	1.03–1.57	0.03	1.23	0.96–1.57	0.11
**Severe neonatal depression (DFM)**	1.7 (19)	1.1 (30)	0.64	0.39–1.03	0.07	0.55	0.29–1.04	0.07
**Admitted to neonatal care (DFM)**	4.4 (52)	4.5 (131)	1.02	0.73–1.41	0.91	1.02	0.69–1.52	0.92
**Preterm births 28**°**- 36**^6^**weeks (DFM)**	12 (145)	10 (169)	0.79	0.62–1.00	0.05	0.79	0.60–1.05	0.10
**FGR < 10 percent (DFM)**	14 (168)	13.5 (391)	0.93	0.77–1.13	0.48	0.97	0.77–1.23	0.82
**Stillbirths (DFM)**	4.2 (50)	2.4 (73)	0.58	0.41–0.84	0.004	0.51	0.32–0.81	0.004
**Normally formed stillbirths (DFM)**	3.9 (46)	2.2 (65)	0.57	0.39–0.83	0.004	0.50	0.31–0.81	0.005
**Stillbirths (rate in total population)**	3.0/1000	2.0/1000	0.67	0.48–0.93	0.02	Not available		
**Normally formed stillbirths (rate in total population)**	2.8/1000	1.8/1000	0.60	0.42–0.85	0.004	Not available		

* Univariate and multivariate logistic regression showing crude (unadjusted) and adjusted odds ratios (OR) with their 95% confidence intervals (CI). † OR adjusted for maternal weight, age, parity, smoking habits and ethnicity (considered as potential confounding factors). DFM: cases of decreased fetal movements.

Among those with DFM, fewer women with a perceived absence of FM waited more than 24 hours, or a perceived decrease for more than 48 hours, before contacting health-care professionals during the intervention. There were no changes over time in the population in potential confounding factors as maternal age, BMI, smoking habits, parity or ethnicity (table 2).

**Table 2:** Descriptive characteristics: women with DFM before and during the intervention

**Characteristics**	Women with DFM before the intervention* n = 1215 n (%)†	Women with DFM during the intervention* n = 3038 n (%)†	***P*‡**
**Age, y mean (SD)**	29.6 (4.9)	29.6 (5.1)	0.625
**BMI, kg/m**^2^			
> 25	386 (36)	1014 (37)	0.474
**Smoking habits**			
Smoking	104 (8.8)	259 (8.9)	0.924
**Maternal age**			
> 35	196 (16.3)	528 (17.6)	0.324
**Primiparity**	559 (51)	1414 (52)	0.490
**Parity**			
Para 0	559 (51)	1414 (52)	0.601
Para 1	372 (34)	878 (33)	
Para 2+	163 (15)	409 (15)	
**Country of origin**			
**Non-western**	221 (20)	510 (18)	0.198

* Data are reported as n (%) unless otherwise noted.

† Denominators vary due to missing values

‡ Chi square tests for the difference between proportions within women with DFM before and during the intervention

At consultations for DFM the use of ultrasound increased while there were no differences in frequency of umbilical artery Doppler examinations. The complete detection rate of FGR following consultations for DFM and subsequent follow up was not captured, only diagnoses set at the initial consultation. This detection rate rose by 83% from 2.4% to 4.4%, p = 0.020 in term (> 36 weeks) pregnancies, and remained unchanged in the preterm (4.5% versus 4.0%, p = 0.604). The use of additional follow up consultations and admissions for induction as a consequence of the initial consultation for DFM was reduced and the number of emergency caesarean sections remained unchanged (table 1). No difference was seen in any other pre-specified secondary outcomes (data not shown).

## Discussion

We found that our interventions combining improved guidelines for management of DFM to health professionals and uniform information on fetal activity to expecting women improved the quality of care and was associated with a reduction of stillbirth rates in our population.

With a large prospective population-based cohort, a low "loss to follow-up" rate, a design with low risk of recruitment bias by outcome, ability to correct for anticipated confounders, large effects on hard outcomes, and confirmation of effects from independent data sources, the assessment of our intervention appear robust. Our quality assessment was conducted as a multi-intervention bundle that aimed to improve the in-hospital management of DFM, including clinical examination, the use of NST and ultrasound, recommended time-lines for health-care professionals, and excluding the use of Doppler. It included general information about fetal activity, recommendations for maternal care-seeking, several rules of thumb for recognizing DFM, and an FM chart as a supportive tool. It also included awareness among health-care professionals, since all obstetricians, general practitioners, community midwives, and others contributing to antenatal care in our population were informed in writing about the ongoing intervention. The exact effect size can only be estimated in randomized trials, which may be challenging and of moderate value unless each individual component of the bundle is tested in a separate trial [1,25]. Implementing only parts of the bundle as a response to the findings of our initial quality-assurance data was not an option in our high-resource setting with a highly educated population. It was considered unacceptable to inform women about DFM without securing professional management of DFM according to the consensus of best practice, and equally unacceptable to perform quality assurance of management of DFM without informing the women to the best of our knowledge about their important role in identifying and reporting DFM.

A much-debated issue is whether women should receive uniform information about FM, and whether this should include formal fetal movement counting (FMC) [25]. This is a method used by the mother to quantify FM, and the source of quantitative definitions of DFM, also called "alarm limits". Two main groups of counting methods exist, using either a "fixed time" or "fixed number" approach. The "Daily Movement Count" [33] reflects 12 hours of maternal FMC through an entire day (i.e., "fixed time"). This method was later modified to shorter and repeated periods of counting [1]. The "Count to ten" or "Cardiff" method uses the time it takes to perceive ten movements (i.e., "fixed number") [34]. The latter method is the most user-friendly, since a shorter time is needed to perform counting for normal pregnancies. This method has also been shown to have the highest compliance and acceptance rates [6,35,36]. While three controlled trials (one randomized) of FMC counting versus no counting has suggested benefit in preventing stillbirths [21,37-39], a large cluster multicentred cluster-randomized controlled trial reported by Grant, Valentin, Elbourne & Alexander in 1989 failed to demonstrate the same benefit using a "Kick Chart" for all pregnancies versus only for risk pregnancies [40]. This is the most referred-to and influential publication on maternal counting, and as such is often cited as evidence against FMC [1,28,41]. However, this trial had several of limitations [1,6]. Of greatest importance is the issue of contamination between the groups through the use of "within-hospital" clusters. The problem of contamination is compounded by the use of Kick Charts for control-group women on the basis of clinical discretion as a part of the trial design. While no difference was shown in the stillbirth rate across the study groups, the overall late-gestation stillbirth rate fell during the study period from 4/1000 to 2.8/1000 [40].

The lowered overall stillbirth rates seen in the observational cohorts and during the cluster-randomized trial might, however, be attributable equally to increased awareness and vigilance, as to the actual FMC methods and alarm limits. Indeed, the cluster-randomized trial used extreme limits (ten movements in 10 hours for two days or no movements for one full day) and based their "count to ten" method on the mother's perception through the day, and not on focused counting while lying down. Thus, the women needed 162 minutes to count ten movements versus the average of 20 minutes reported in focused counting [20,21,42]. Despite the extreme nature of such limits, they are still widely used [43]. There is no evidence that formal FMC with their fixed alarm limits are superior to maternal common sense, no evidence to support the introduction of such counting in any total population, and no rationale to perform trials using the existing alarm limits of FMC [25]. Better tools to identify the pregnancy at risk by assessing FM patterns are needed, and they will have to be individually adjusted to identify change, not fixed levels, to reflect what pregnant women are actually reporting. However, the routine of daily FMC in the third trimester could provide additional vigilance in the individual pregnancy, and help the expectant mother to identify significant changes. Our information highlighted the importance of the woman's subjective perception of a significant and sustained reduction in FM as the primary indicator of DFM, and a cause to seek professional help. We suggested daily FMC only as a tool to aid monitor FM, and guided the woman with "ten FM within 2 hours" as a secondary rule of thumb in situations where she felt in doubt.

The goal of antepartum fetal surveillance is to exclude imminent fetal jeopardy, identify risk pregnancies and aid in the prevention of adverse outcomes [27]. Controlled trials of management of DFM are lacking [7,12]. While the behaviour of health-care professionals related to the time of referral or examination remained unchanged during the intervention, the use of ultrasound changed. This was in accordance with the consensus-based guidelines of our study [12] indicating that NST and ultrasound examination were the most useful tools for fetal surveillance in DFM, and consistent with the evidence for antepartum testing in other risk pregnancies [12,44-46]

A weakness of the assessment of the intervention is that there are no codes for visits due to consultations for DFM in the electronic medical files of the Norwegian hospital system. Thus, no validation of the completeness of registrations of cases of DFM was possible with the anonymous of files used. Bias may have been introduced through the health professionals' inclusion of cases either by registration fatigue over time or increased enthusiasm by the general awareness caused by the intervention. This would, however, not affect the results on stillbirth rates in the total population, and not the outcomes among cases with DFM. Only a systematically skewed registration towards more or less severe cases of DFM would affect these results, and our design separating inclusion from outcome registration would counteract such effects. An additional weakness of the intervention is that we do not have the overall caesarean section and induction rate in the total population. However, it is unlikely that there would be any increase in the total population as the caesarean section rate following consultations for DFM remained unchanged and the induction rate was reduced. Clinical quality interventions in a population are based on the existing imperfections found by prior data collections of quality indicators, as we have demonstrated in our community. The results may thus not be directly transferable to other populations. Yet, reports from a variety of locations suggest that significant variability in the management of DFM and of information given to expecting women is a wide-spread quality issue in obstetric care [2,5,12,29].

There may be concerns that such a quality improvement intervention would increase interventions and iatrogenic injuries. This was not observed in our population. There was no increase in consultations for DFM, and, while no formal cost analysis was performed, it is likely that the added cost of ultrasound was compensated by reduced use of admissions for induction and repeated follow up consultations. Increased confidence in the adequacy of the management plan could have contributed to this change in behavior among health-care professionals.

## Conclusion

Improved quality of management of DFM and uniform information to improve the value of the existing "self-screening" of fetal activity was associated with a reduction in stillbirth rates in our population. For further improvements, new and individually adjusted definitions of DFM are needed, as well as randomized controlled trials to determine the optimal management and information to pregnant women with DFM. Further research is required to identify optimal methods for detecting important reductions in FM if DFM is to be an effective screening tool for adverse pregnancy outcomes.

## Abbreviations

BMI: Body mass index; DFM: Decreased fetal movements; FGR: Fetal growth restriction; FM: Fetal movements; FMC: Fetal Movement Counting; NST: Non-stress test.

## Competing interests

The authors declare that they have no competing interests.

## Authors' contributions

JVHT: Design of the study, collection, analysis and the interpretation of data, writing and finalizing the manuscript. ES: Design of the study, collection, analysis and the interpretation of data and revising the manuscript. BSP: Design of the study, the interpretation of data and revising the manuscript. PEB: Design of the study, the interpretation of data and revising the manuscript. VF: Design of the study, the interpretation of data and revising the manuscript. RF: Design of the study, the interpretation of data and revising the manuscript. JFF: Design of the study, collection, analysis and the interpretation of data, writing and revising the manuscript. All authors have approved the final version of the manuscript.

## Pre-publication history

The pre-publication history for this paper can be accessed here:



## Supplementary Material

Additional file 1 **Kicks Count**. Kicks Count brochure, Norwegian version. A brochure of information aiming to increase maternal awareness and vigilance to significant decreases in fetal activity, and to aid health promoting behavior. The brochure was provided as a part of the routine information given to women at the standard ultrasound assessment at 17–19 weeks in Norway as a part of the quality improvement intervention. Click here for file

Additional file 2 **Kicks Count**. Kicks Count brochure, English version. A brochure of information aiming to increase maternal awareness and vigilance to significant decreases in fetal activity, and to aid health promoting behavior. The brochure was provided as a part of the routine information given to women at the standard ultrasound assessment at 17–19 weeks in Norway as a part of the quality improvement intervention. Click here for file

Additional file 3 **Kicks Count**. Kicks Count brochure, Arabic version. A brochure of information aiming to increase maternal awareness and vigilance to significant decreases in fetal activity, and to aid health promoting behavior. The brochure was provided as a part of the routine information given to women at the standard ultrasound assessment at 17–19 weeks in Norway as a part of the quality improvement intervention. Click here for file

Additional file 4 **Kicks Count**. Kicks Count brochure, Turkish version. A brochure of information aiming to increase maternal awareness and vigilance to significant decreases in fetal activity, and to aid health promoting behavior. The brochure was provided as a part of the routine information given to women at the standard ultrasound assessment at 17–19 weeks in Norway as a part of the quality improvement intervention. Click here for file

Additional file 5 **Kicks Count**. Kicks Count brochure, Somali version. A brochure of information aiming to increase maternal awareness and vigilance to significant decreases in fetal activity, and to aid health promoting behavior. The brochure was provided as a part of the routine information given to women at the standard ultrasound assessment at 17–19 weeks in Norway as a part of the quality improvement intervention. Click here for file

Additional file 6 **Kicks Count**. Kicks Count brochure, Urdu version. A brochure of information aiming to increase maternal awareness and vigilance to significant decreases in fetal activity, and to aid health promoting behavior. The brochure was provided as a part of the routine information given to women at the standard ultrasound assessment at 17–19 weeks in Norway as a part of the quality improvement intervention. Click here for file
