# Correction: Reduction of late stillbirth with the introduction of fetal movement information and guidelines - a clinical quality improvement

**DOI:** 10.1186/1471-2393-10-49

**Published:** 2010-09-02

**Authors:** Julie Victoria Holm Tveit, Eli Saastad, Babill Stray-Pedersen, Per E Børdahl, Vicki Flenady, Ruth Fretts, J Frederik Frøen

**Affiliations:** 1 Division of Obstetrics and Gynecology, and Centre for Perinatal Research, Rikshospitalet University Hospital, University of Oslo, Medical Faculty, Norway; 2 Norwegian Institute of Public Health, Division of Epidemiology, Oslo, Norway; 3 Akershus University College, and University of Oslo, Medical faculty, Norway; 4 Department of Obstetrics and Gynecology, Haukeland University Hospital, Bergen, Norway; 5 Institute for Clinical Medicine, Section for Gynaecology and Obstetrics, University of Bergen; 6 Department of Obstetrics and Gynecology, University of Queensland, Mater Mothers' Hospital, South Brisbane, Australia; 7 Brigham and Women's Hospital, Division of Maternal-Fetal Medicine, Harvard Medical School, Boston, MA

## Abstract

We have performed a full cross-validation of this clinical Femina data collection against the routinely collected data of the Medical Birth Registry of Norway to validate the estimates of reduced mortality in the total population. The original estimate of fewer deaths during the intervention with OR 0.7 remains virtually unchanged for the original data collection.

The validation procedures revealed inaccuracies in data from the Medical Birth Registry of Norway for a partial comparison with mortality outside the study area, and we here correct this comparison. We present new, corrected and cross-validated data. Despite comparability issues, the most robust and cross-validated estimates confirm similar estimates of reduced mortality during the quality improvement intervention.

## Introduction: Comparison with registry data

This article is based on the clinical Femina data collection - independent of the Medical Birth Registry of Norway (MBRN). However, a partial comparison with the population outside the cohort was included, using MBRN data (1).

In accordance with the study protocol of 2005, we aimed to compare our Femina data with MBRN data for the full cohort for an independent validation of stillbirth rates in the total population. The complete data set for Norway for 2007, needed for this final comparison, was released by the MBRN on December 13, 2009. Upon receipt of these complete data we found discrepancies with the data our project had previously received from the MBRN and published (1). The MBRN performed an inquiry into the two data deliveries, and on February, 17, 2010, the MBRN issued a public report which confirmed that the previous data delivery to our project was inaccurate.

We deeply regret this, and wish to correct the original article accordingly and provide new and validated data.

## Correction

From the section Data collection, the following sentence describes the data found to be inaccurate, and should be discarded: "*In addition to the registrations by our study protocol, the numbers of births and stillbirths from our population were obtained from the Medical Birth Registry in Norway to assess overall trends in stillbirth, for the most updated period available: April 2005 to December 2006*."

From the third paragraph of the Results section, the following sentence is based on the inaccurate data, and should be discarded: "*Independent data from the Medical Birth Registry in Norway, confirmed that the stillbirth rate in our total cohort of births was comparable to the rest of Norway in the baseline observation (OR 1.06; 95% CI 0.70-1.65, p = 0.73), and significantly lower during the intervention period (OR 0.64; 95% CI 0.47-0.87, p = 0.005)*."

## Limitations in comparisons of Femina data and MBRN data

There was a dual capture of deaths in the Femina study. Primarily, deaths were registered retrospectively by clinical study site coordinators (midwife or obstetrician) reporting births, deaths and causes of death monthly from the clinical logs and hospital records. All hospitals provided monthly reports. In addition, women presenting with a complaint of decreased fetal movements were captured prospectively, prior to the registration of outcome (1).

Notification of all births to the MBRN is compulsory in Norway. However, missed or erroneous key variables leading to missing capture of cases may occur in any registry.

Femina and MBRN data differ in some aspects. 1) Femina did not register cases born after ≥ 28 weeks if death occurred prior to 28 weeks. Time of intrauterine death is not reported to the MBRN. 2) In Femina the clinicians reported the best estimate of gestational age (combining clinical assessment, last menstrual period, ultrasound screening and autopsies). The MBRN is based on the LMP and ultrasound alone. 3) Femina included immediate neonatal deaths in the delivery room, which would by definition not be captured as a stillbirth in the MBRN.

In their report of February 17, 2010, the MBRN find that gestational age alone is insufficient to track third trimester stillbirths due to missing data on gestation. For comparisons with the Femina data they therefore report cases of ≥ 28 weeks of gestation and a birth weight ≥ 1000 grams, or one of these criterions if the other is missing (Cat. 28). The MBRN also reports that the completeness of stillbirth reports increase with gestation; this is also our experience. With the existing limitations for comparisons at the limits around 28 weeks and 1000 grams, the MBRN suggest to report cases of ≥ 32 weeks and 1500 grams (Cat. 32) to minimize bias in comparisons. We agree that this improves comparability, and probably represent the most robust data for comparing the point estimates (odds ratios), despite having less statistical power due to smaller groups and thus wider confidence intervals.

## New data from the MBRN and cross-validation with Femina data

We found some discrepancies between the MBRN and Femina in the number of deaths registered. Prior to intervention, the MBRN registered 47 deaths in Cat. 28, while Femina registered significantly more cases, altogether 56. During the intervention, both registered 92. Due to the concerns this raised, the Norwegian Institute of Public Health (NIPH), owners of both the Femina and MBRN data, combined Femina and MBRN registrations on day and hospital of birth, birth weight and gestational age to compare case by case. The probability of identical details for all four variables in separate cases is negligible in our setting - e.g. two deaths on the same day in the same delivery unit only occurred once in our two-year study, and their gestation and weight differed. Cases on which both registries agreed were deemed to be validated by each other.

In total, there were 33 unique Cat. 28 cases only found in one of the datasets. The hospitals in question were requested to re-confirm these cases to the NIPH. Two duplicates in the Femina material were found by this procedure: The dual prospective and retrospective capture of stillbirths in Femina, described above, lead to two stillbirths being reported twice from different hospitals. The two duplicate reports did not mention that the stillbirth had occurred in another hospital, and slight differences in the details reported made them go unrecognized.

A cross-validated dataset may be the most robust estimate available, compensating for underreporting to both datasets by including all deaths registered in any of the two. Validation identified 46 deaths prior to vs. 78 during intervention in Cat. 32, and 55 deaths vs. 102 in Cat. 28.

Overall, for stillbirths ≥ 28 weeks/1000 grams, 10% were not found in the MBRN, and 7% were not found in Femina. For the MBRN, this does not exclude the possibility that they had been reported in some form, but neither gestation nor birth weight identified them as deaths in any of these categories.

## Analyses of the cross-validated data, Femina data and MBRN data

Removing the two duplicates from the Femina data provides an essentially identical estimate of the original significant association with lower mortality in the total population with OR 0.7 (table 1). In the subset Cat. 32 the estimates of OR 0.7 is found to be identical in both the Femina data, the MBRN data, as well as in the cross-validated data combining Femina and MBRN, and the widened confidence interval a natural consequence of the smaller subset from the total material. In the cross-validated data the mortality rates are 2.4/1000 prior to vs. 1.7/1000 during intervention.

**Table 1:** Odds ratios (OR) and 95% confidence intervals (CI) for all comparisons of mortality associated with the intervention period, both within the Femina cohort (actual study), outside the study area (trends unrelated to study), and between the study area and the rest of Norway, stratified by the data sources available for the comparison.

*Comparison*	*Femina area: Intervention period vs. pre-intervention period*		*Rest of Norway: Intervention period vs. pre-intervention period*		*Femina area vs. rest of Norway prior to intervention period*		*Femina area vs. rest of Norway during intervention period*	
*Group & data source*	OR	95% CI	OR	95% CI	OR	95% CI	OR	95% CI
								
*Original comparison*								
Femina data	0.69	0.50 - 0.96	-	-	-	-	-	-
MBRN data	-	-	-	-	-	-	-	-
Cross-validated data	-	-	-	-	-	-	-	-
								
*≥ 32 weeks or 1500 g*								
Femina data	0.74	0.50 - 1.08	-	-	-	-	-	-
MBRN data	0.71	0.48 - 1.05	1.11	0.71 - 1.73	1.16	0.71 - 1.88	0.74	0.53 - 1.04
Cross-validated data	0.72	0.50 - 1.03	-	-	-	-	-	-
								
*≥ 28 weeks or 1000 g*								
Femina data	0.72	0.52 - 1.01	-	-	-	-	-	-
MBRN data	0.83	0.58 - 1.18	1.07	0.72 - 1.59	1.05	0.68 - 1.63	0.82	0.61 - 1.10
Cross-validated data	0.79	0.57 - 1.09	-	-	-	-	-	-

In the subset Cat. 28 we find support for the expectations, discussed above, that the clearest differences in data collection and reporting, are found in the lowest gestational ages. With Cat. 32 estimates being identical in all three datasets, the one fifth of deaths occurring between 28 and 32 weeks account for the discrepancies. During the intervention, reporting of these early deaths to Femina remained unchanged (increased by 9%, 7 vs. 18 cases among 19035 vs. 44967 births) while reporting to the MBRN increased by 80% (4 vs. 17 cases). As a result, the MBRN finds an estimate of OR 0.8 where Femina finds OR 0.7 in Cat. 28.

For analyses of mortality rates outside our study area, only MBRN data is available. In Cat. 32 these indicate more deaths in the Femina area than in the rest of Norway prior to intervention with OR 1.2, while this is reversed during intervention to OR 0.7 (table 1). As noted above, the intervention in the Femina area was associated with OR 0.7 while in the rest of Norway there was an increased number of deaths with OR 1.1 in this period. In Cat. 28, again, the estimate in the MBRN is OR 0.8 rather than 0.7.

## Conclusion: Support of original estimates, but more studies needed

The validation of MBRN and Femina data show that neither had optimal robustness - 10% and 7% of deaths were not identified, respectively. Thorough validation using independent data collections was needed to identify two duplicates. Yet, the reproduction of identical estimates of OR 0.7 among deaths in Cat. 32 in Femina data, MBRN data and cross-validated data, lend significant support to the validity of the study's original data collection and results. The discrepancy produced by including deaths between 28 and 32 weeks questions whether there was truly more deaths in this group during the intervention (as the MBRN data may suggest), or whether the rate was unaffected and discrepancy is due to data collection/comparability issues (as the comparison of Femina and MBRN data may suggest). In an intervention increasing stillbirth awareness among health professionals, an increased proportion of early gestation deaths being reported to the MBRN is not surprising. In a prolonged quality improvement project like ours, "registration fatigue" would not be surprising either.

In taking all possible comparisons into account, we find odds ratios of 0.69, 0.71, 0.72, 0.72, 0.74, 0.74, 0.79, 0.82 and 0.83, mostly at borderline significance levels. It therefore seems prudent to estimate an association between the intervention and mortality in the range of OR 0.7 - 0.8. The precise effect of optimal information to pregnant women about decreased fetal movements, and the optimal management of complaints for decreased fetal movements, remains to be identified in randomized controlled trials.

We have reviewed the commentaries in light of our findings. The MBRN data were not directly questioned by Dr. Salvesen, however, he did compare with the MBRN and found reasons for concern over numbers that apparently demonstrated the opposite of the actual results of the study (2). Dr. Salvesen should be commended for his interest in the study and for acting on such concerns. The published data indicated that a comparison based solely on gestational age in the MBRN was valid and helpful, which is regrettable.

## Pre-publication history

The pre-publication history for this paper can be accessed here:

http://www.biomedcentral.com/1471-2393/10/49/prepub
