# Supplementary material for: Reduction of late stillbirth with the introduction of fetal movement information and guidelines – a clinical quality improvement
Source: BMC Pregnancy Childbirth. 2009 Jul 22;9:32. doi: 10.1186/1471-2393-9-32 (PMC2734741; doi:10.1186/1471-2393-9-32)
Supplement: Additional file 1 — Kicks Count. Kicks Count brochure, Norwegian version. A brochure of information aiming to increase maternal awareness and vigilance to significant decreases in fetal activity, and to aid health promoting behavior. The brochure was provided as a part of the routine information given to women at the standard ultrasound assessment at 17–19 weeks in Norway as a part of the quality improvement intervention. [file 1471-2393-9-32-S1.pdf]

Teller du spark kan du bidra til forskningen

Husk at barnet skal sparke hver dag!

Bli kjent med barnet ditt!

Kjenn etter hver dag!

... Spark er mer enn bare kos ...

Tell trivselen

www.telltrivselen.no

BLI KJENT MED BARNET DITT!

Du har nå kommet så langt i svangerskapet at du kanskje har kjent barnet sparke. Svangerskapskontrollene vil hjelpe deg med å følge barnets trivsel, men det er du som mamma som kan bli best kjent med det før det blir født. Det barnet sier med sparkingen, er viktig!

Etter fødselen vil du bruke mye tid til å ta deg av barnet. Vi oppfordrer deg til å bruke litt tid hver dag i svangerskapet til å bli kjent med barnet ved å kjenne etter bevegelser. Her finner du informasjon om hva det betyr når det sparker, og veiledning i hvordan du kan følge med på at barnet ditt trives.

Det er en god vane å sette av tid til å bli kjent med barnet!

HVA SIER SPARKINGEN OM TRIVSEL?

I morkaken overføres alt barnet trenger. Så lenge forsyningene er gode, sparker barnet. Blir det lite forsyninger fra morkaken, eller barnet blir sykt, må det spare på kreftene for å kunne fortsette å vokse - sparkingen avtar. Røyker du, vil både morkake og barnet rammes, og sparkingen vil avta. Om svikten i morkaken blir alvorlig, hemmes veksten, og barnet kan bli sykt eller skadet. Da kan det også tåle fødselen dårligere. Dette skjer sjelden dersom barnet sparker normalt!

Av og til er det stille. Det som betyr noe, er dersom den vanlige sparkingen avtar mye. Når du kjenner den vanlige rytmen på livet der inne, er dette et tegn på at barnet har det bra. At du kjenner etter sparkene, bidrar også til at du knytter deg nærmere barnet ditt. Det er derfor det er fint å telle trivselen!

Derfor teller vi trivselen!

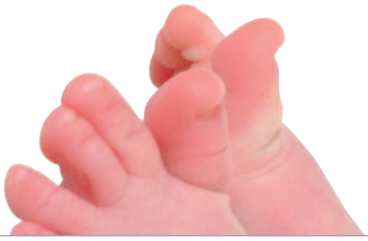

Når det sparker i magen - hva betyr det?

HVA GJØR BARNET DER INNE?

Barnet ditt vil være aktivt i hele svangerskapet. Det det gjør mest av, er pustebevegelser du ikke kjenner. Slik utvides lungene, mens det øver seg til tiden etter fødselen. Av og til får det hikke, som du kjenner som regelmessige små rykk.

Barnet utfører både store og små bevegelser inne i magen din. Små gripebevegelser, suging på tommelen eller bøying og strekking av fingre og tær kjenner du nok ikke. De aller fleste spark og dytt barnet gjør der inne, kjenner du derimot godt i siste del av svangerskapet. Større kroppsbevegelser er også ofte tydelige og kan komme når du endrer stilling fra stående til liggende. Da blir barnet snudd litt, og kan svare med å flytte litt på seg, slik det ofte dytter tilbake om du dytter litt på det.

AV OG TIL ER DET STILLE...

Barnet ditt sover mange ganger i løpet av dagen og da er det stille. Søvnperiodene blir lengre utover i svangerskapet, men selv nær termin sover barnet sjelden mer enn en time om gangen. De fleste er mest aktive på kvelden, og mange også tidlig om morgenen.

Det kan være stor forskjell fra barn til barn på hvor mye og kraftig de "sparker" – med spark mener vi her alle typer bevegelser. Barn som sparker mye, er ofte mer aktive etter fødselen også. Det er ikke kjent å være forskjell på gutter og jenter.

Noen mødre kjenner sparkene dårligere enn andre. Hvis morkaken ligger på fremsiden av livmoren eller du er overvektig, kjenner du mindre. Du kan øve deg på å kjenne etter spark samtidig som du ser om magen beveger seg. Du kjenner sparkene best når du ligger, og minst når du står, går eller er opptatt med noe.

Foto: Per Oscar Skjellnan • www.peroscar.no
Design: Marianne Bratt Ricketts • www.mariannedesign.no

Har du spørsmål? Se www.telltrivselen.no

TELLE TRIVSELEN?

Å kjenne etter spark hver dag er en god vane. Sparkeskjemaet er et enkelt hjelpemiddel som du kan bruke hvis du vil, og som gir deg og jordmoren/legen din en oversikt over barnets sparking. Skjemaet gjør det lettere å se hva som er normalt for ditt barn. Selv om sparking er viktig for alle, er sparkeskjemaet best egnet etter uke 28 i svangerskapet, og dersom du ikke har tvillinger.

HVORDAN DELTA I FORSKNINGEN?

Fyller du ut sparkeskjemaet og leverer det etter fødselen, bidrar du også til viktig forskning om hvordan man kan kjenne igjen svangerskap med behov for ekstra hjelp på grunnlag av sparkeaktiviteten. Om du ønsker å delta i slik forskning, finner du informasjon om dette på folderens bakside.

HVORDAN TELLER MAN?

Start å telle trivselen når du er kommet til uke 28 i svangerskapet. Skriv inn datoen du begynner og terminen din på skjemaet.

Tell til 10 – ta tiden fra du kjenner det første sparket (så du vet at barnet er våkent). Alle bevegelser gjelder som "spark", men hikke skal du ikke telle! Flere bevegelser samtidig gjelder for ett "spark". Det går fortest om du ligger/sitter avslappet og kjenner ordentlig etter. Om barnet sover, er det lov å vekke det med litt klapping/dytting på magen eller ved at du drikker noe kaldt.

Sett et kryss i ruten på skjemaet for tiden det tok å telle 10 spark og hvilken dag det er.

Tell omtrent på samme tid hver dag - hvis du kan. Velg en tid på døgnet når det passer deg best å sette av litt tid og når du vet at barnet pleier å være aktivt. Start tellingen innen de samme to timene hver dag. Skriv på skjemaet i hvilket tidsrom du pleier å telle.

Gjør du det slik, vil de fleste oftest bruke under 15 minutter på å telle trivselen.

Hvordan fylle ut telleskjemaet

Kryss av med blå/sort penn i ruten. Etter at du er ferdig med å telle til 10 spark setter du et kryss i ruteskjema hvor du angir hvor lang tid dette tok.

Eksempel: Brukte du for eksempel 30 minutter før du kjente 10 spark, setter du et kryss i skjemaet i ruten 26-30 minutter.

Dersom det tok 1 time og 5 minutter før du kjente 10 spark, setter du et kryss i ruten 61-65 minutter.

Minutter	Dag 1	Dag 2	Dag 3	Dag 4	Dag 5	Dag 6	Dag 7
0-5							
6-10							
11-15							
16-20							
21-25	X						
26-30		X					
31-35							
36-40							
41-45							
46-50							
51-55							
56-60							
61-65				X			
66-70							
71-75							
76-80							
81-85							
86-90							
91-95							
96-100							
101-105							
106-110							
111-115							
116-120							
Hvis du brukte mer enn 2 timer, skriv ned tiden du brukte							

Minutter

Minutter

Hvor mye skal barnet sparke – og hva om det avtar?

Etter å ha brukt sparkeskjemaet i en uke eller to, vil du nok se at sparkingen varierer litt fra dag til dag, men at dagene stort sett ligner på hverandre. Slik vil det fortsette for et barn som trives, selv om måten du føler bevegelser på endrer seg under svangerskapet. Det viktigste er at du ikke opplever stor og vedvarende nedgang i forhold til normal aktivitet for barnet ditt! Sparkeskjemaet hjelper deg å se dette.

Hvis du er bekymret for barnet ditt, bør du uansett grunn søke råd og hjelp. Er du bekymret fordi barnet ditt gradvis sparker mindre ettersom ukene går, bør du ta med deg sparkeskjemaet til neste svangerskapskontroll.

I noen tilfeller bør du ta direkte kontakt med fødeavdelingen:

- Hvis barnet ditt ikke sparker en dag, skal du aldri vente til neste dag.
- Hvis barnet sparker stadig mindre i løpet av dagen/dager, og du kjenner "lite liv".

Er du i tvil om hva som er "lite liv", bør du vite at det er svært sjelden at et friskt barn sparker mindre enn 10 spark i løpet av to timer du vet den pleier å være aktiv. Har du følt at det har sparket så lite hele dagen, bør du kontakte fødeavdelingen. Om du ikke vet om det har sparket så lite i tiden før du startet dagens telling, bør du følge med. Sørg da for å telle igjen innen de neste 12 timene og kontakt fødeavdelingen dersom resultatet gjentar seg.

Jeg begynte å telle i dag (dato, dd.mm.åå) __.__.__ Jeg har termin (dd.mm.åå) __.__.__ Jeg pleier å telle mellom kl: __.__ og kl: __.__

MINUTTER

5 min per boks

	UKE 28							UKE 29							UKE 30							UKE 31							UKE 32							UKE 33							UKE 34						
UKEDAGER	Dag 1	Dag 2	Dag 3	Dag 4	Dag 5	Dag 6	Dag 7	Dag 1	Dag 2	Dag 3	Dag 4	Dag 5	Dag 6	Dag 7	Dag 1	Dag 2	Dag 3	Dag 4	Dag 5	Dag 6	Dag 7	Dag 1	Dag 2	Dag 3	Dag 4	Dag 5	Dag 6	Dag 7	Dag 1	Dag 2	Dag 3	Dag 4	Dag 5	Dag 6	Dag 7	Dag 1	Dag 2	Dag 3	Dag 4	Dag 5	Dag 6	Dag 7	Dag 1	Dag 2	Dag 3	Dag 4	Dag 5	Dag 6	Dag 7
START TELLING 0-5																																																	
6-10																																																	
11-15																																																	
16-20																																																	
21-25																																																	
26-30																																																	
31-35																																																	
36-40																																																	
41-45																																																	
46-50																																																	
51-55																																																	
56-60																																																	
61-65																																																	
66-70																																																	
71-75																																																	
76-80																																																	
81-85																																																	
86-90																																																	
91-95																																																	
96-100																																																	
101-105																																																	
106-110																																																	
111-115																																																	
116-120																																																	
Hvis du brukte mer enn 2 timer, skriv ned tiden du brukte																																																	

UKEDAGER | MINUTTER

	UKEDAGER	0-5	6-10	11-15	16-20	21-25	26-30	31-35	36-40	41-45	46-50	51-55	56-60	61-65	66-70	71-75	76-80	81-85	86-90	91-95	96-100	101-105	106-110	111-115	116-120	Over 2t? Skriv ned tiden du brukte
UKE 42	Dag 7																									
	Dag 6																									
	Dag 5																									
	Dag 4																									
	Dag 3																									
	Dag 2																									
	Dag 1																									
UKE 41	Dag 7																									
	Dag 6																									
	Dag 5																									
	Dag 4																									
	Dag 3																									
	Dag 2																									
	Dag 1																									
UKE 40	Dag 7																									
	Dag 6																									
	Dag 5																									
	Dag 4																									
	Dag 3																									
	Dag 2																									
	Dag 1																									
UKE 39	Dag 7																									
	Dag 6																									
	Dag 5																									
	Dag 4																									
	Dag 3																									
	Dag 2																									
	Dag 1																									
UKE 38	Dag 7																									
	Dag 6																									
	Dag 5																									
	Dag 4																									
	Dag 3																									
	Dag 2																									
	Dag 1																									
UKE 37	Dag 7																									
	Dag 6																									
	Dag 5																									
	Dag 4																									
	Dag 3																									
	Dag 2																									
	Dag 1																									
UKE 36	Dag 7																									
	Dag 6																									
	Dag 5																									
	Dag 4																									
	Dag 3																									
	Dag 2																									
	Dag 1																									
UKE 35	Dag 7																									
	Dag 6																									
	Dag 5																									
	Dag 4																									
	Dag 3																									
	Dag 2																									
	Dag 1																									

DELTA I FORSKNING OM SAMMENHENGENE MELLOM SPARKEAKTIVITET OG HELSE?

Vi vil gjerne be deg delta i forskningen ved å levere inn denne folderen etter at du har født barnet ditt - tvillinger skal ikke være med. Dette er uavhengig av om du har brukt sparkeskjemaet ditt eller ei.

HVEM OG HVORFOR?

I regi av Folkehelseinstituttet, Perinatalt forskningssenter ved Rikshospitalet, Sanitetskvinnene og Landsforeningen uventet barnedød gjøres det nå undersøkelser om sammenhengene mellom barns sparkeaktivitet i magen, og deres fremtidige helse. Vi vet at alvorlig nedgang eller opphør av sparkeaktivitet er forbundet med risiko for sykdom og død, men vet lite om hvordan dette og annen informasjon om barnets sparkeaktivitet i magen kan brukes til å fremme barns helse på kort og lang sikt.

DET ER HELT FRIVILLIG Å DELTA

For forskningens del er det viktig at flest mulig leverer inn denne folderen før de forlater føde- / barselavdelingen etter fødsel. Folderen er verdifull for forskningen selv om du ikke skulle ha brukt sparkeskjemaet eller ikke vil gi noen opplysninger om deg selv. Er du under 18 år, bør du spørre dine foresatte.

DU KAN BIDRA TIL FORSKNINGEN PÅ EN AV TRE MÅTER AVHENGIG AV HVOR MYE INFORMASJON DU GIR:

1. Hvis du deltar i Den norske mor & barn undersøkelsen, kan du samtykke ved å la informasjonen fra folderen din knyttes til undersøkelsen. Du kan angi ditt personnummer og signere her:

Signatur: ______________________ Personnummer: ☐☐ ☐☐ ☐☐ ☐☐☐☐☐

2. Du kan gi samtykke til at informasjonen fra folderen din knyttes til Medisinsk fødselsregister ved å angi ditt fødsels-og personnummer og signere her:

Signatur: ______________________ Personnummer: ☐☐ ☐☐ ☐☐ ☐☐☐☐☐

3. Vil du være anonym fyller du ut opplysningene nederst på denne siden og gir ditt samtykke ved å krysse av her: ☐

ER DET TRYGT Å DELTA?

Folkehelseinstituttet har ansvaret for fortrolighet og sikkerhet for dine opplysninger. Instituttet har konsesjon fra Datatilsynet og tilråding fra Forskningsetisk komité til å ta vare på informasjon om deg og barnet ditt.
Så snart Folkehelseinstituttet har registrert dine opplysninger og knyttet deg til informasjonen du har gitt samtykke til, vil din informasjon anonymiseres. Dette betyr at folderen din destrueres og all identifiserende informasjon fjernes før forskere får se informasjonen.

HVOR SKAL JEG LEVERE FOLDEREN?

Det finnes tydelig merkede postkasser på alle føde- og barselavdelinger. Spør personalet om du er i tvil.

I hvilken svangerskapsuke fødte du?..................... ☐☐
(uke 40 er termin)

HVORDAN FØDTE DU?

Vanlig fødsel (vaginal).................................... ☐
Keisersnitt som var planlagt .... ☐
Keisersnitt underveis i fødselen .... ☐
Barnets kjønn Gutt ☐ Jente ☐
Barnets fødselsvekt ☐☐☐☐ gram

HVORDAN GIKK DET MED BARNET?

Barnet var friskt .... ☐
Barnet ble innlagt på nyfødtavdeling/barneavdeling ☐

Din alder .... ☐☐ år
Din vekt før svangerskapet .... ☐☐☐ hele kg
Din høyde .... ☐☐☐ hele cm

Hvor mange barn har du født før? ☐☐

Røykte du den siste måneden av dette svangerskapet?
Nei .... ☐
Av og til ... ☐ ☐☐ sigaretter per uke
Daglig ..... ☐ ☐☐ sigaretter per dag

Har du annet morsmål enn norsk? Ja ☐ Nei ☐
Hvis JA, hvilket språk? ☐☐☐☐☐☐☐☐☐☐

---

folkehelseinstituttet

Rikshospitalet - Radiumhospitalet HF

LANDSFORENINGEN UVENTET BARNEDØD

BLI MED I TREKNINGEN!!

Leverer du inn folderen din kan du være med i en månedlig trekning av **Libero/Natusan produkter** til en verdi av **kr. 1000,-**

Vil du være med i trekningen? JA ☐ NEI ☐

Navn .... tel ....

Kontaktadresse:
Julie Holm Tveit/Frederik Frøen/
Eli Saastad
FEMINA
Nasjonalt folkehelseinstitutt, EPAM
Postboks 4404 Nydalen
N-0403 Oslo
Tel: +47 23 07 02 26
+47 23 41 94
Fax: +47 23 40 82 52
