# Supplementary material for: Reduction of late stillbirth with the introduction of fetal movement information and guidelines – a clinical quality improvement
Source: BMC Pregnancy Childbirth. 2009 Jul 22;9:32. doi: 10.1186/1471-2393-9-32 (PMC2734741; doi:10.1186/1471-2393-9-32)
Supplement: Additional file 2 — Kicks Count. Kicks Count brochure, English version. A brochure of information aiming to increase maternal awareness and vigilance to significant decreases in fetal activity, and to aid health promoting behavior. The brochure was provided as a part of the routine information given to women at the standard ultrasound assessment at 17–19 weeks in Norway as a part of the quality improvement intervention. [file 1471-2393-9-32-S2.pdf]

GET TO KNOW YOUR BABY!

You have come so far into your pregnancy that you might have already felt your baby kick. Your pregnancy check-up will help you keep an eye on your baby's well-being, but you the mother are the one who can "know" your baby best before it is born. What the baby is telling you with its kicks is important!

After the birth you will be spending a lot of time caring for your baby. We encourage you to devote a little time each day during your pregnancy to get to know your baby by its movements. Here you will find information about what kicks mean as well as some tips on how you can check your baby's well-being.

It's a good habit to set aside time to get to know your baby!

WHAT DO THE KICKS SAY ABOUT WELL-BEING?

Your baby gets everything it needs from you through the placenta. As long as the supplies are good the baby will kick. If the placenta does not supply enough or the baby becomes ill, it must save energy to continue to grow, so there are fewer kicks. If you smoke, both the placenta and your baby are affected, and there will be less kicking. If the placenta becomes very weak, growth is inhibited, and your baby could become seriously ill or injured. Your baby also may have trouble during birth. This seldom happens, though, if your baby is kicking normally!

Sometimes your baby is calm. It is important that you notice, though, when it kicks much less than usual. When you feel the normal life rhythm in there, that is a sign your baby is fine. By feeling for the kicks you are connecting with your baby. That's why it's good to count kicks!

That's why we count kicks!

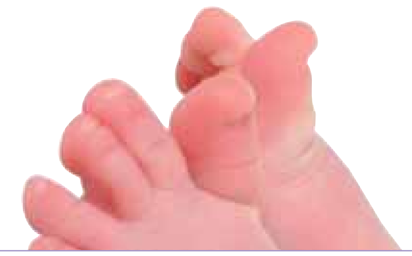

When it kicks in the womb - what does this mean?

WHAT IS YOUR BABY DOING IN THERE?

Your baby will be active during the entire pregnancy. Mostly, it makes movements to expand its lungs. Your baby is practicing for life outside the womb. From time to time it will have hiccups, which you will feel as small twitches.

Your baby also will make minor and more pronounced movements in your womb. You probably will not feel small gripping movements, or when your baby sucks its thumb or bends and stretches its fingers and toes. However, you will feel most of your baby's kicks and nudges inside you during the last part of the pregnancy. More pronounced body movements also are easy to notice and can come, for example, when you change from a sitting to a lying position. When you shift and need to adjust a little bit, so does your baby.

SOMETIMES IT'S JUST CALM...

Your baby will sleep many times in the course of a day. During these times it does not move at all. Its periods of sleep last longer as the pregnancy goes on, but even as you approach the expected date of delivery your baby will seldom sleep more than an hour at a time. Most babies are especially active in the evening, though some prefer moving around early in the morning.

There can be great variations among healthy babies as to how often and how hard they "kick." Kicking includes all types of movements. Research shows girls kick as often as boys. Babies who kick a lot in the womb are also more active after birth.

Some mothers have more trouble feeling the kicks than others. If the placenta is on the front side of the womb, or if you are overweight, you will feel the kicks less. You can practice feeling for kicks when you check to see if your stomach moves. You feel the kicks best when you are lying down, and least when you stand, walk or are busy.

Photo: Per Oscar Skjellnan • www.peroscar.no
Design: Marianne Bratt Ricketts • www.mariannedesign.no

If you have any questions, go to www.telltrivselen.no

KICKS COUNT

Feeling for kicks every day is a good habit. Filling out the kick count form is an easy way to give you and your midwife/doctor an overview of your baby's kicking. The form makes it easier to see what is normal for your baby. Even though kicking is important for all expectant mothers, the kick count form is most helpful starting in the 28th week of pregnancy for mothers carrying one child, not multiples.

If you fill in the form and submit it after giving birth, you will be contributing to important research on how doctors can recognize pregnancies that need extra help on the basis of kicks. You will find information on how to take part in this research on the back page.

HOW DO YOU COUNT?

Start counting the kicks in the 28th week of your pregnancy. Write in the date you start keeping count and your expected delivery date on the form.

You should count how long it takes your baby to kick 10 times, starting with the first kick (so you know your baby is awake). All movements count as a "kick" but don't count hiccups. Several movements at the same time count as one "kick." The quickest way to do this is to relax, lie or sit down and concentrate on feeling for kicks. If your baby is asleep, you can wake it with a little clapping or gentle push on your stomach or by drinking something cold.

When you're finished counting record the time in the boxes provided on the form.

Count at approximately the same time of day every day - if you can. Choose a time of day when you know you have time to spare and when your baby usually is active - preferably in the morning before you get up. Start counting within the same two-hour period every day and be sure to write on the form whatever hours you choose.

It will take most mothers less than 15 minutes to count kicks this way.

How to fill in the form:

Use a black/blue pen to mark the box to show how long it took before you counted 10 kicks.

For example: If 30 minutes went by before you counted 10 kicks, you mark the box 26-30 minutes.

If it took 1 hour and 5 minutes until you counted 10 kicks, mark the box for 61-65 minutes.

Minutes	Day 1	Day 2	Day 3	Day 4	Day 5	Day 6	Day 7
0-5							
6-10							
11-15							
16-20							
21-25	X						
26-30		X					
31-35							
36-40							
41-45							
46-50							
51-55							
56-60							
61-65			X				
66-70							
71-75							
76-80							
81-85							
86-90							
91-95							
96-100							
101-105							
106-110							
111-115							
116-120							
Over 2 hours							

Minutes

Minutes

How much should the baby kick - and what if the number of kicks declines?

After having used the kick count form for a week or two, you will probably see that the kicking varies from day to day, but that for the most part the days appear to be similar. This will continue to be the case for a child who has good well-being, even if the way you feel the movements changes during the pregnancy. The most important thing is to note when there is a major and lasting reduction in the normal activity of your baby. The kick count form will help you see this.

If you are worried about your baby, you should ask for help and advice regardless of the reason. If you are worried because your child gradually kicks less as the weeks go by, you should take your kick count form to your next pregnancy check-up.

In some cases you should contact the maternity ward directly:

- If your baby does not kick one day. If this happens NEVER wait till the next day.
- If your baby kicks less and less in the course of a day/days and you feel "too little activity."

If you are in doubt as to what constitutes "to little activity", it may be helpful to know that very rarely does a healthy child kick fewer than 10 times in the course of two hours when it is usually active. If you feel it has kicked very little the whole day, you should contact the maternity ward. If you are unsure of your baby's activity on a given day, recount within 12 hours and contact the maternity ward if the result is repeated.

You can use the folder as a holder for your "helsekort for gravide".
The "Helsekort" as well as any lose papers can be fastened here.

Note: Tick the box with a pen

I start counting today (date, dd.mm.yy) ☐☐.☐☐.☐☐ My due date is (dd.mm.yy) ☐☐.☐☐.☐☐ I normally count between (time): ☐☐.☐☐ and ☐☐.☐☐

MINUTES

5 minutes per box (0-60) — 5 minutes per box (61-120)

WEEKDAYS	Day 1	Day 2	Day 3	Day 4	Day 5	Day 6	Day 7	Day 1	Day 2	Day 3	Day 4	Day 5	Day 6	Day 7	Day 1	Day 2	Day 3	Day 4	Day 5	Day 6	Day 7	Day 1	Day 2	Day 3	Day 4	Day 5	Day 6	Day 7	Day 1	Day 2	Day 3	Day 4	Day 5	Day 6	Day 7	Day 1	Day 2	Day 3	Day 4	Day 5	Day 6	Day 7	Day 1	Day 2	Day 3	Day 4	Day 5	Day 6	Day 7
START COUNTING 0-5																																																	
6-10																																																	
11-15																																																	
16-20																																																	
21-25																																																	
26-30																																																	
31-35																																																	
36-40																																																	
41-45																																																	
46-50																																																	
51-55																																																	
56-60																																																	
61-65																																																	
66-70																																																	
71-75																																																	
76-80																																																	
81-85																																																	
86-90																																																	
91-95																																																	
96-100																																																	
101-105																																																	
106-110																																																	
111-115																																																	
116-120																																																	
Over 2 hours																																																	
	WEEK 28							WEEK 29							WEEK 30							WEEK 31							WEEK 32							WEEK 33							WEEK 34						

NB. Make a cross in the square using a pen

When you have filled in the form send it or hand it in to:

	WEEKDAYS	MINUTES 0-5	6-10	11-15	16-20	21-25	26-30	31-35	36-40	41-45	46-50	51-55	56-60	61-65	66-70	71-75	76-80	81-85	86-90	91-95	96-100	101-105	106-110	111-115	116-120	Over 2 hours
WEEK 42	Day 7																									
	Day 6																									
	Day 5																									
	Day 4																									
	Day 3																									
	Day 2																									
	Day 1																									
WEEK 41	Day 7																									
	Day 6																									
	Day 5																									
	Day 4																									
	Day 3																									
	Day 2																									
	Day 1																									
WEEK 40	Day 7																									
	Day 6																									
	Day 5																									
	Day 4																									
	Day 3																									
	Day 2																									
	Day 1																									
WEEK 39	Day 7																									
	Day 6																									
	Day 5																									
	Day 4																									
	Day 3																									
	Day 2																									
	Day 1																									
WEEK 38	Day 7																									
	Day 6																									
	Day 5																									
	Day 4																									
	Day 3																									
	Day 2																									
	Day 1																									
WEEK 37	Day 7																									
	Day 6																									
	Day 5																									
	Day 4																									
	Day 3																									
	Day 2																									
	Day 1																									
WEEK 36	Day 7																									
	Day 6																									
	Day 5																									
	Day 4																									
	Day 3																									
	Day 2																									
	Day 1																									
WEEK 35	Day 7																									
	Day 6																									
	Day 5																									
	Day 4																									
	Day 3																									
	Day 2																									
	Day 1																									

DO YOU WANT TO TAKE PART IN RESEARCH ON THE CONNECTION BETWEEN A BABY'S KICKING AND ITS HEALTH?

We would like to ask you to take part in this research by handing in this folder after you have given birth to your baby –- if you delivered one baby — regardless of whether you have used the kick counting form or not.

WHO AND WHY?

The Norwegian Institute of Public Health, Rikshospitalet University Hospital, Norwegian Women´s Public Health Organisation and unexpected Child Death Society of Norway are studying the connection between a baby's kicking in the womb and its future health. We know that steep reduction in or cessation of kicking is connected with a risk of illness or death, but we know little about how this and other information about the child's kicking in the womb can be used to promote the child's health in the short and long term.

PARTICIPATION IS VOLUNTARY AND YOU CAN CONTRIBUTE A LOT OR A LITTLE

The more women who submit their folders, the better it is for the research. So please submit yours after giving birth but before leaving the maternity ward. The folder is valuable to the research even if you have not used the kick counting form or do not want to give any information about yourself. But the more information you give, the more valuable your contribution. If you are under 18 years of age, you should ask your parents/guardian for permission to participate. Remember, you choose how much you want to contribute so:

DO YOU WANT TO BE ANONYMOUS?

Then fill in the information at the bottom of this page and give your consent by marking here: ☐

CAN YOU CONTRIBUTE MORE?

You can give your consent to allow the information from your folder to be connected with the medical birth register by giving your date of birth and national identity number (fødsels- og personnummer) and signing here:

Signature: ______________________ National identity number ☐☐ ☐☐ ☐☐ ☐☐☐☐☐

OR

If you are participating in the Norwegian mother & child study, you can give your consent to allow the information from your folder to be connected with the medical birth register by giving your date of birth and national identity number and signing here:

Signature: ______________________ National identity number ☐☐ ☐☐ ☐☐ ☐☐☐☐☐

IS IT SAFE TO PARTICIPATE? WILL MY IDENTITY BE PROTECTED?

The Norwegian Institute of Public Health is responsible for the confidentiality and security of your information. The institute is licensed by the Data Inspectorate and approved by the Research Ethics Committee to store information about you and your child. As soon as the institute has registered all your information, your folder will be destroyed and your data made anonymous. Researchers will see only the anonymous data.

WHERE SHOULD I HAND OVER THE FOLDER?

There are clearly marked boxes in all maternity wards. Ask one of the hospital staff if you are in doubt.

I don't want to give any information.................. ☐

In which week of the pregnancy was the delivery?... ☐☐
(week 40 is the expected delivery week)

HOW DID YOU DELIVER?

Normal delivery (vaginal)................................... ☐
Planned Caesarean .... ☐
Caesarean decided during labour .... ☐
Emergency Caesarean .... ☐
Child's gender Boy ☐ Girl ☐
Child's weight at birth ☐☐☐☐ grams

HOW DID IT GO WITH THE CHILD?

The child was healthy.. ☐
The child was admitted to the postnatal/children's ward ☐
The child died .... ☐

How old are you?.................................. ☐☐ yrs
Your weight before pregnancy .... ☐☐☐ whole kg
Your height .... ☐☐☐ whole cm
How many children have you already given birth to? ☐☐

Did you smoke the last month of this pregnancy?
No ☐
Sometimes ☐ ☐☐ cigarettes per week
Daily ☐ ☐☐ cigarettes per day

Do you have another native language other than Norwegian? Yes ☐ No ☐
If yes, which language? ☐☐☐☐☐☐☐☐☐☐

---

folkehelseinstituttet
Rikshospitalet – Radiumhospitalet HF

Thanks for your help!

Translated by Tolketjenesten in Oslo.
November 2005

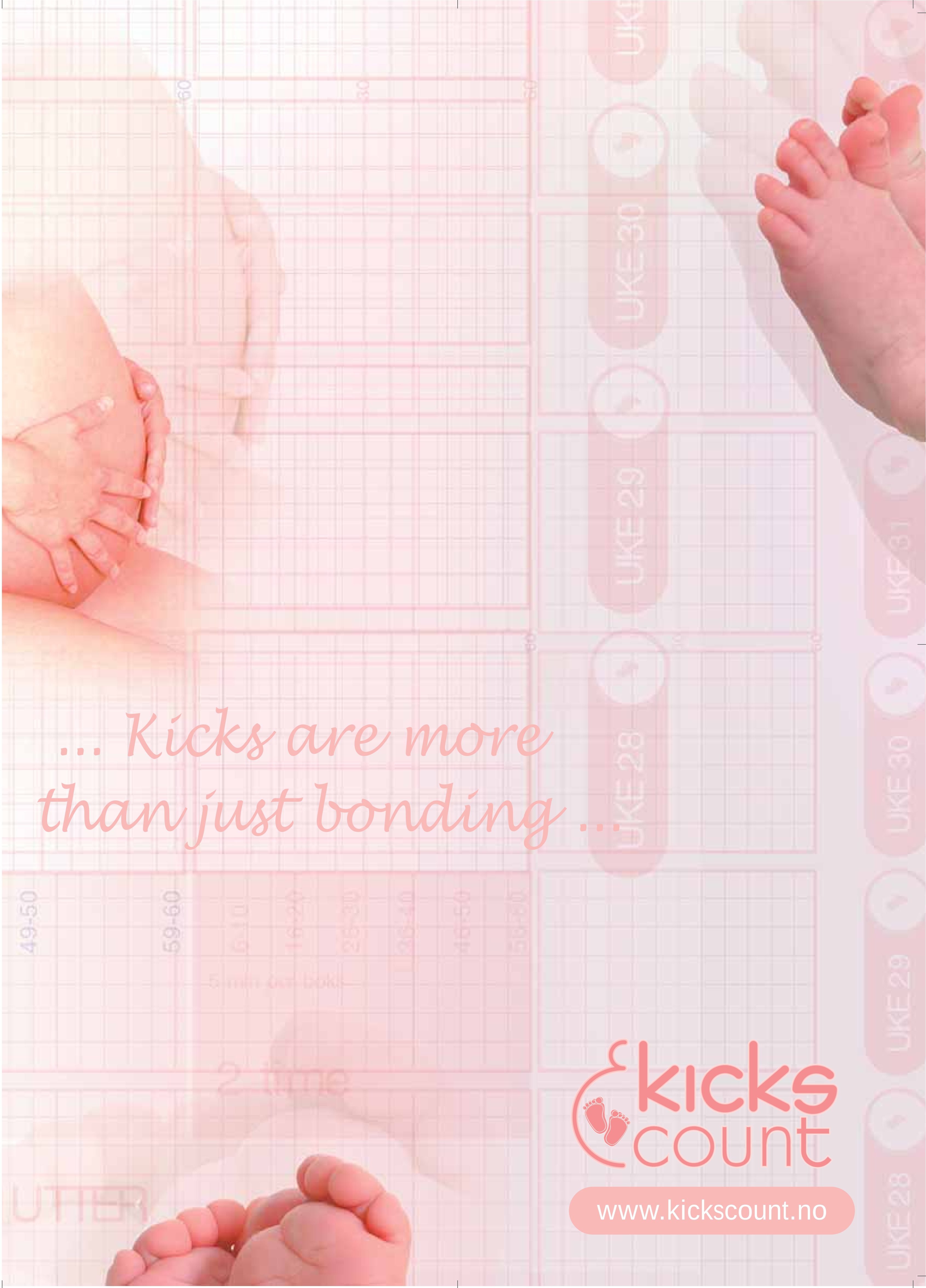
… Kicks are more
than just bonding …
kicks
count
www.kickscount.no
