# Supplementary material for: Reduction of late stillbirth with the introduction of fetal movement information and guidelines – a clinical quality improvement
Source: BMC Pregnancy Childbirth. 2009 Jul 22;9:32. doi: 10.1186/1471-2393-9-32 (PMC2734741; doi:10.1186/1471-2393-9-32)
Supplement: Additional file 3 — Kicks Count. Kicks Count brochure, Arabic version. A brochure of information aiming to increase maternal awareness and vigilance to significant decreases in fetal activity, and to aid health promoting behavior. The brochure was provided as a part of the routine information given to women at the standard ultrasound assessment at 17–19 weeks in Norway as a part of the quality improvement intervention. [file 1471-2393-9-32-S3.pdf]

ملاحظة: استخدمي قلم حبر في وضع اشارة الضرب

لقد بدأت العد هذا اليوم (التاريخ، اليوم.الشهر.السنة) ☐☐.☐☐.☐☐

لقد تعودت على العد بين الساعة: ☐☐.☐☐ و الساعة: ☐☐.☐☐

إن موعد ولادتي المتوقع (التاريخ، اليوم. الشهر. السنة) ☐☐.☐☐.☐☐

أيام الاسبوع

اليوم 1 · اليوم 2 · اليوم 3 · اليوم 4 · اليوم 5 · اليوم 6 · اليوم 7 (under each week)

ابدئي العد

دقائق

		الاسبوع 28	الاسبوع 29	الاسبوع 30	الاسبوع 31	الاسبوع 32	الاسبوع 33	الاسبوع 34
الساعة الأولى (5 دقائق لكل مربع)	0-5							
	6-10							
	11-15							
	16-20							
	21-25							
	26-30							
	31-35							
	36-40							
	41-45							
	46-50							
	51-55							
	56-60							
الساعة الثانية (5 دقائق لكل مربع)	0-5							
	6-10							
	11-15							
	16-20							
	21-25							
	26-30							
	31-35							
	36-40							
	41-45							
	46-50							
	51-55							
	56-60							
	أكثر من ساعتين							

عد الضربات؟

إن تحسس ضربات الطفل كل يوم، تعتبر عادة جيدة، إن الاستمارة الخاصة بالضربات هي من أبسط وسائل المساعدة التي تعطيك أنت والقابلة القانونية والطبيب نظرة عامة عن ضربات الطفل. وبالتالي يصبح من السهل معرفة ماهو الطبيعي بالنسبة لطفلك. بالرغم من أن ضربات الطفل مهمة للجميع، ولكن استمارة الضربات يمكن الاعتماد عليها بعد الاسبوع 28 من الحمل، وذلك في حال أنك لاتحملين بتوأم.
املئي استمارة الضربات أثناء حملك، وسلميها بعد ولادتك، تكونين بذلك قد ساعدت أيضاً في الأبحاث المهمة الجارية حول كيفية معرفة المرء بالحمل وتقدير احتياجه إلى مساعدة إضافية بناء على نشاط الضربات. إذا أردت المشاركة في مثل هذه الأبحاث، يمكنك الحصول على معلومات خاصة بذلك من خلال الملحق المثبت خلف الورقة.

كيف يعد المرء؟

ابدئي بعدد الضربات عندما تكونين في الاسبوع 28 من الحمل، سجلي في الاستمارة تاريخ بدايتك في العد وكذلك تاريخ ولادتك المتوقع.

عدي عشرة ضربات - احسبي الوقت من بدء احساسك بالضربة الأولى (عندما تتأكدين بأن طفلك مستيقظ).
جميع الحركات تحسب كضربات، ولكنك لن تعدين الحازوقة. تعد الحركات المتراكبة كضربة واحدة. يمكن أن تتم الحركات بسرعة وتتحسسيها بشكل واضح سواء كنت في وضع الاستلقاء أو الجلوس أو الراحة. يحق لك في حال أن الطفل كان نائماً أن تجعليه يستيقظ إما بالضغط قليلاًعلى البطن أو بدفعه أو بشرب شراباً بارداً.

ضعي إشارة ضرب في المربع الصغير في الاستمارة للوقت الذي استخدمتيه في عد 10 ضربات وفي أي يوم تم ذلك.

ابدئي العد في نفس الوقت تقريباً من كل يوم، وللمدة التي تستطيعينها. اختاري وقتاً مناسباً لك خلال اليوم وخصصي منه فترة قصيرة تعرفين فيها ان طفلك يكون عادة نشيطاً خلالها، ويفضل أن يكون ذلك في الصباح قبل نهوضك. ابدئي العد ضمن نفس الساعتين من كل يوم. سجلي في الاستمارة في أي وقت تبدئين فيه العد عادة.
إذا قمت بالعد بهذا الشكل فإنك ستستعملين أقل من 15 دقيقة لعد الضربات مثل غالبية الحوامل.

كيف يمكنك إملاء استمارة العد:

ضعي اشارة ضرب في المربع، بقلم أسود أو أزرق. بعد أن تكوني قد انتهيت من عد 10 ضربات، ضعي اشارة ضرب في المربع الذي يبين الزمن الذي استغرقه عمل ذلك

مثال:

لقد استغرق عد 10 ضربات 30 دقيقة، ضعي اشارة ضرب في المربع 26 - 30 دقيقة.

في حال استغرق عد 10 ضربات ساعة و5 دقائق، ضعي اشارة ضرب في حقل الساعة الثانية ساعتين في المربع 0-5 دقائق.

	اليوم 1	اليوم 2	اليوم 3	اليوم 4	اليوم 5	اليوم 6	اليوم 7
0-5							
6-10							
11-15							
16-20							
21-25	X						
26-30		X					
31-35							
36-40							
41-45							
46-50							
51-55							
56-60							

0-5			X				
6-10							
11-15							
16-20							
21-25							
26-30							
31-35							
36-40							
41-45							
46-50							
51-55							
56-60							
**أكثر من ساعتين**							

كم هي عدد الضربات التي يقوم بها الطفل - وماذا لو تباطأت؟

يمكنك وبعد استخدامك لاستمارة الضربات لمدة اسبوع أو اسبوعين، ملاحظة أن الضربات تختلف من يوم إلى آخر، ولكنها تتشابه بشكل عام في جميع الأيام، وتستمربنفس الشكل لطفل يشعر بالارتياح، على الرغم من أن طريقة شعورك بالحركة قد تتغير خلال فترة حملك به. من المهم ألا تشعرين بضعف كبير ومستمر في حركة طفلك بالمقارنة مع نشاطه المعتاد. إن استمارة الضربات تساعدك على كشف ذلك.

إذا كنت قلقة بشأن طفلك، يستحسن بك مهما كانت أسباب قلقك، طلب المساعدة والنصيحة. أما إذا كان قلقك ناتجاً عن شعورك بضعف تدريجي في حركة طفلك مع تقدم أسابيع الحمل، فعليك عندها أخذ استمارة الضربات هذه عند ذهابك لمتابعة فحص الحمل.

يستحسن بك الاتصال مباشرة بقسم الولادة وذلك في الحالات التالية:

- إذا لم يتحرك طفلك يوماً كاملاً، عليك ألا تنتظري أبداً إلى اليوم الذي يليه.
- إذا كانت ضربات الطفل ضعيفة بشكل مستمر خلال اليوم/ أيام، وتشعرين بضعف في الحركة.

إذا كنت في شك حول مامعنى "ضعف في الحركة" ، فعليك أن تعلمي أنه من النادر جداً لطفل معافى أن يضرب أقل من 10 ضربات خلال ساعتين وتعرفين أنه نشيط في العادة. يستحسن بك الاتصال بقسم الولادة، وذلك إذا شعرت أن ضرباته كانت ضعيفة طوال اليوم. تابعي حركة الطفل، خاصة إذا كنت لاتعرفين فيما إذا كانت حركته ضعيفة في الفترة التي لم تبدئي فيها العد اليومي بعد. اسعي للعد ثانية في الـ12 ساعة القادمة واتصلي بقسم الولادة في حال ان النتائج كانت متماثلة.

إلى "helsekort for gravide"

تعرفي على طفلك

لقد قطعت الآن شوطاً طويلاً في حملك، ومن المرجح أنك بدأت تشعرين بضربات الطفل. تساعدك مراجعات الحمل الطبية في متابعة مدى ارتياح الطفل، ولكنك كأم تبقين الوحيدة التي بإمكانها معرفة مدى ارتياحه حتى قبل ولادته. إن ما يعبر عنه الطفل بضرباته يعتبر مهماً.

سوف تقضين وقتاً طويلاً بعد الولادة من أجل الاعتناء بطفلك. لذا نحثك على استخدام بعض من وقتك يومياً للتعرف على طفلك أثناء حملك وذلك من خلال متابعتك لحركته. سوف تجدين هنا معلومات وافيه عن معنى الضربات، وكذلك التوجيهات لمعرفة كيفية متابعتها وذلك للتأكد من مدى ارتياح طفلك.

إن تخصيص وقت للتعرف على الطفل هو عادة جيدة.

كيف تخبر الضربات عن مدى الارتياح؟

ينتقل بواسطة المشيمة كل مايحتاجه الطفل منك. يبقى الطفل يتحرك لطالما أن هذا الانتقال يتم بشكل جيد. أما إذا كان هذا الانتقال ضعيفاً أو إذا الطفل مريضاً فتضعف عندها حركته، وذلك لأنه يتوجب عليه اختزان طاقاته من أجل استخدامها في النمو. إذا كنت تدخنين، فإن ذلك يؤثر على الطفل والمشيمة بشكل مباشر وبالتالي تضعف حركة الطفل. إذا أصبح تقاعس المشيمة كبيراً، يؤخر هذا عملية النمو، ويمكن أن يؤدي إلى مرض خطير عند الطفل أو إصابته بأذى. كما يصبح تحمله للولادة ضعيفاً، إن ماذكر يحدث نادراً وخاصة إذا كانت ضربات الطفل طبيعية.

يعم الهدوء في بعض الأحيان، ولكن هذا لايعني شيئاً، إلا إذا كان هناك تباطؤ كبير في ضربات الطفل المعتادة. عندما تشعرين بدلائل حياة متوازنة في داخلك، فهذه إشارة بأن طفلك وضعه جيد. إن شعورك بضربات طفلك يساعدك على التقرب منه أكثر. لهذا فمن الجيد أن تعدي ضرباته لتتابعي ارتياحه.

لهذا السبب نعد ضربات الطفل

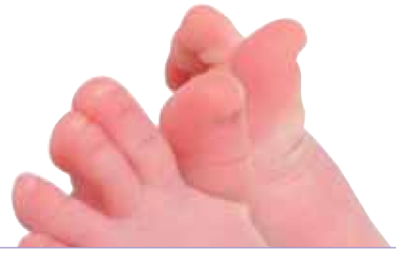

ماذا يعني أن يضرب الطفل في أحشائك؟

ماذا يفعل الطفل في الداخل؟

يبقى الطفل نشيطاً طوال فترة الحمل. إن أكثر مايفعله هو حركات تنفس لاتشعرين بها. وهكذا تتوسع رئتاه من خلال التمارين التي يقوم بها ليهيء نفسه لما بعد الولادة. يصاب الطفل في بعض الأحيان بالحازوقة والتي تشعرين بها على شكل رعشة بسيطة ومنتظمة.

يقوم الطفل بإجراء حركات صغيرة وكبيرة في أحشائك. إن الحركات الصغيرة كمص سبابته أو انحنائه أومد أصابع يديه وقدميه هي حركات لاتشعرين بها، أما معظم الضربات والدفع الذي يقوم به الطفل في الداخل تشعرين به بشكل واضح وذلك في الفترات الأخيرة من الحمل. تكون معظم حركات جسمه الكبيرة واضحة، ويمكن أن تحدث مثلاً: عندما تغيرين من وضعية الوقوف إلى الاستلقاء، عندها يميل الطفل ويحرك نفسه قليلاً رداً على حركتك هذه، كما يقوم غالباً بالدفع عندما تدفعيه قليلاً.

أحياناً يعم الهدوء

ينام طفلك مرات عديدة في اليوم، عندها يعم الهدوء بشكل تام. تطول فترات نوم الطفل مع التقدم في الحمل. ولكن نادراً ما ينام أكثر من ساعة في كل مرة وذلك عند اقتراب موعد ولادته. يكون معظمهم أكثر نشاطاً في المساء، والكثيرون منهم أيضاً يكونون أكثر نشاطاً في الصباح الباكر.

يمكن أن يكون هناك اختلاف كبير بين طفل وآخر في حجم وقوة ضرباته، ونقصد بالضربات هنا جميع أشكال الحركات. الأطفال الذين يتحركون أكثر من غيرهم يكونون في الغالب أكثر نشاطاً بعد الولادة أيضاً. ومن الممكن أن يتمتع الجميع بنفس القدرمن العافية. لم يعرف بعد بأن هناك اختلاف بين الذكور والإناث.

إن احساس بعض الأمهات بالحركة أضعف من غيرهن. فإذا كان توضع المشيمة أمام الرحم أو إذا كانت الأم بدينة يصبح الاحساس بالحركة أضعف. يمكنك التدرب على الاحساس بالحركة في نفس الوقت الذي ترين فيه بطنك يتحرك. إن شعورك بالضربات يكون أكثر عندما تستلقين وأقل عندما تقفين أو تمشين أو عندما تكونين مشغولة بشيء ما.

الصورة : Per Oscar Skjellnan

التصميم : Marianne Bratt Ricketts • www.mariannedesign.no

هل عندك سؤال؟ انظري في الموقع: www.telltrivselen.no

[arabisk]

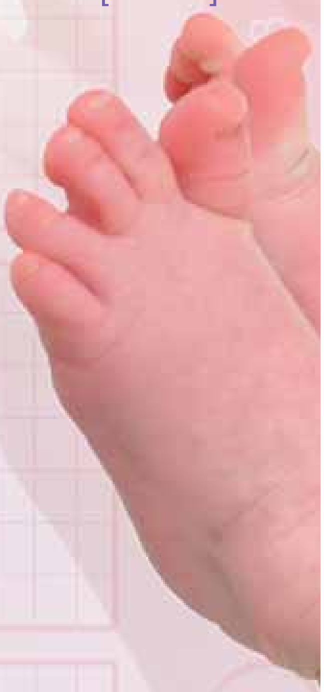

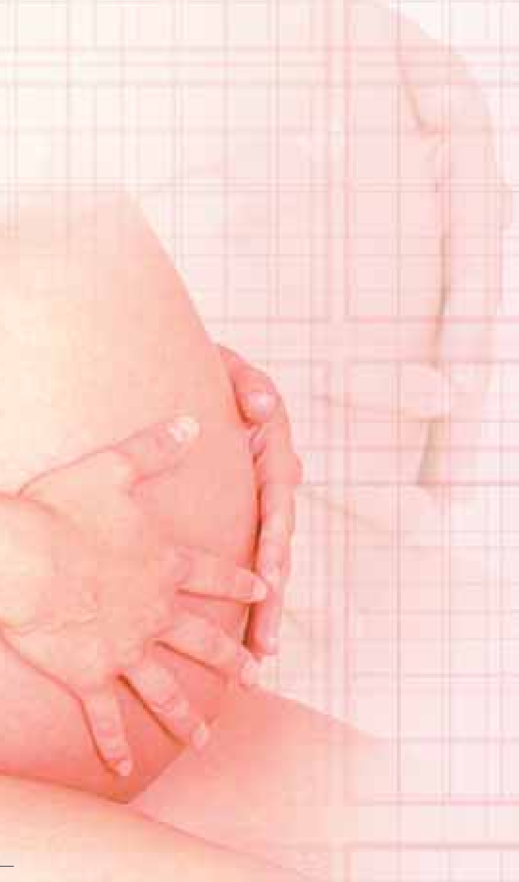

...ضربات الطفل هي ليست
للتسلية فقط...

kicks count

www.kickscount.no

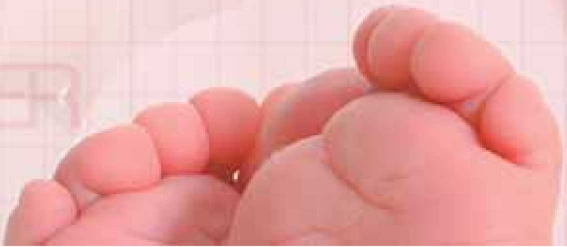

شاركي في البحث القائم حول العلاقة بين نشاط ضربات الجنين وصحته.

نرغب في دعودتك للمشاركة في البحث، وذلك عن طريق تسليم هذه الاستمارة بعد ولادتك، وذلك في حال أنك ولدت طفلاً واحداً، ومشاركتك هذه غير مرتبطة باستخدامك لاستمارة الضربات أو عدمه.

من ولماذا؟

تجرى حالياً وبإشراف المعهد الصحي الشعبي (Folkehelseinstituttet) ، و المركز الطبي للبحوث المتعلقة بفترة ماقبل وبعد الولادة، و العيادة النسائية في مشفى (Rikshospitalet Radiumhospitalet HF) ، و جمعية النساء المسعفات، أبحاث حول العلاقة بين نشاط ضربات الطفل في البطن ومستقبله الصحي. نحن نعلم أن الضعف الكبير في ضربات الطفل أوتوقفها مرتبط بخطورة تعرضه للأمراض أو وفاته. ولكن معرفتنا قليلة حول كيفية استخدام ذلك وبمساعدة معلومات أخرى متعلقة بنشاط ضربات الطفل في البطن للوصول إلى طفل معافى على المدى القريب والبعيد.

المشاركة طوعية، ويمكنك المساهمة بذلك إما قليلاً أو كثيراً .

من المهم ولصالح البحث المجرى أن تقوم أكثرهن بتسليم هذه الاستمارة وذلك قبل مغادرتهن قسم الولادة والنفاس.
إن لهذه الاستمارة قيمة كبيرة في البحث، حتى وإن لم تستخدمي استمارة الضربات أو أنك لاترغبين في إعطاء معلومات عن نفسك. تزداد هذه القيمة بقدر مساهمتك.
يستحسن بك إذا كنت لم تبلغي الثامنة عشر من العمر استشارة أولياء أمرك. يمكنك وكلأتي تحديد مدى مساهمتك الشخصية في البحث :

هل تريدين أن تبقي غير معلنة؟

دوني المعلومات في أسفل هذه الصفحة، وامنحي موافقتك وذلك بوضع اشارة ضرب هنا : ☐

هل بإمكانك المساهمة أكثر ؟

يمكنك منح الموافقة على جعل المعلوملات الواردة في استمارتك ترتبط مباشرة ببيانات الولادة الطبية، وذلك من خلال تدوين تاريخ ميلادك ورقمك الشخصي والتوقيع هنا:

الرقم الشخصي: ☐☐ ☐☐ ☐☐ ☐☐☐☐☐

التوقيع: ____________________

أو

إذا كنت ستشاركين في البحث النرويجي وهو " الأم والطفل "، يمكنك عندها منح موافقتك لجعل المعلومات الواردة في استمارتك تستخدم في البحث وذلك بكتابة رقمك الشخصي والتوقيع هنا:

الرقم الشخصي: ☐☐ ☐☐ ☐☐ ☐☐☐☐☐

التوقيع: ____________________

هل المشاركة آمنة؟

تقع على عاتق معهد الصحة الشعبي مسؤولية الحفاظ على سرية المعلومات الخاصة بك. يملك المعهد إجازة خاصة من قبل دائرة مراقبة الحاسوب، كما تقدم له الارشادات من قبل لجنة البحوث الخاصة بالأخلاق المهنية وذلك من أجل المحافظة على المعلومات الخاصة بك وبطفلك.
يقوم معهد الصحة الشعبي في أقرب وقت يتم فيه تسجيل المعلومات الخاصة بك، وربطك بالمعلومات التي وافقت على منحها، بجعل تلك المعلومات غير معلنة. هذا يعني أن استمارتك تتلف وجميع المعلومات الخاصة بهويتك تمحى وذلك قبل اطلاع الباحثين عليها.

أين بإمكاني تسليم الاستمارة؟

هناك صندوق معنون في جميع أقسام الولادة والنفاس. اسألي العاملين هناك إذا لم تجديه.

لاأريد اعطاء أية معلومات .... ☐

في أي من أسابيع حملك تمت ولادتك؟ .... ☐☐

(الاسبوع رقم 40 هو اسبوع الولادة)

كيف تمت ولادتك؟

ولادة طبيعية (مهبلية)........................ ☐

ولادة قيصرية مخطط لها.................. ☐

ولادة قيصرية تم تقريرها أثناء الولادة ..... ☐

ولادة قيصرية سريعة .... ☐

جنس المولود ذكر ☐ أنثى ☐

وزن الطفل عند الولادة ☐☐☐☐ غرام

كيف سارت الأمور مع الطفل؟

كان الطفل معافى.. ☐

تم إدخال الطفل إلى قسم حديثي الولادة /قسم الأطفال.... ☐

توفي الطفل .... ☐

عمرك...................................... ☐☐سنة

وزنك قبل الحمل .... ☐☐☐ بعدد صحيح من الكغ

طولك............................ ☐☐☐ بعدد صحيح من السم

كم عدد الأطفال الذين أنجبتيهم مسبقاً ☐☐

هل قمت بالتدخين في الشهر الأخير من حملك هذا؟

لا .... ☐

بعض الأحيان ..... ☐ ☐☐ سيجارة في الاسبوع

يومياً .... ☐ ☐☐ سيجارة في اليوم

هل لغتك الأم هي لغة أخرى غير النرويجية؟.......... نعم ☐ لا ☐

إذا كان الجواب نعم، فما هي اللغة؟ ☐☐☐☐☐☐☐☐☐☐

---

folkehelseinstituttet

Rikshospitalet – Radiumhospitalet HF

شكراً للمساعدة

Oversatt ved Tolketjenesten i Oslo.
November 2005

أيام الاسبوع
دقائق

ملاحظة: استخدمي قلم حبر في وضع اشارة الضرب

عندما تنتهين من إملاء الاستمارة أرسليها على العنوان:

		الساعة الأولى												الساعة الثانية												
	أيام الاسبوع	0-5	6-10	11-15	16-20	21-25	26-30	31-35	36-40	41-45	46-50	51-55	56-60	0-5	6-10	11-15	16-20	21-25	26-30	31-35	36-40	41-45	46-50	51-55	56-60	أكثر من ساعتين
الاسبوع 42	اليوم 7																									
	اليوم 6																									
	اليوم 5																									
	اليوم 4																									
	اليوم 3																									
	اليوم 2																									
	اليوم 1																									
الاسبوع 41	اليوم 7																									
	اليوم 6																									
	اليوم 5																									
	اليوم 4																									
	اليوم 3																									
	اليوم 2																									
	اليوم 1																									
الاسبوع 40	اليوم 7																									
	اليوم 6																									
	اليوم 5																									
	اليوم 4																									
	اليوم 3																									
	اليوم 2																									
	اليوم 1																									
الاسبوع 39	اليوم 7																									
	اليوم 6																									
	اليوم 5																									
	اليوم 4																									
	اليوم 3																									
	اليوم 2																									
	اليوم 1																									
الاسبوع 38	اليوم 7																									
	اليوم 6																									
	اليوم 5																									
	اليوم 4																									
	اليوم 3																									
	اليوم 2																									
	اليوم 1																									
الاسبوع 37	اليوم 7																									
	اليوم 6																									
	اليوم 5																									
	اليوم 4																									
	اليوم 3																									
	اليوم 2																									
	اليوم 1																									
الاسبوع 36	اليوم 7																									
	اليوم 6																									
	اليوم 5																									
	اليوم 4																									
	اليوم 3																									
	اليوم 2																									
	اليوم 1																									
الاسبوع 35	اليوم 7																									
	اليوم 6																									
	اليوم 5																									
	اليوم 4																									
	اليوم 3																									
	اليوم 2																									
	اليوم 1																									

الساعة الأولى
الساعة الثانية
