# Supplementary material for: Reduction of late stillbirth with the introduction of fetal movement information and guidelines – a clinical quality improvement
Source: BMC Pregnancy Childbirth. 2009 Jul 22;9:32. doi: 10.1186/1471-2393-9-32 (PMC2734741; doi:10.1186/1471-2393-9-32)
Supplement: Additional file 4 — Kicks Count. Kicks Count brochure, Turkish version. A brochure of information aiming to increase maternal awareness and vigilance to significant decreases in fetal activity, and to aid health promoting behavior. The brochure was provided as a part of the routine information given to women at the standard ultrasound assessment at 17–19 weeks in Norway as a part of the quality improvement intervention. [file 1471-2393-9-32-S4.pdf]

Çocuğunuzu tanıyın!

Hamileliğiniz o kadar ilerledi ki, belki de çocuğun tekmelediğini hissetmeye başladınız. Hamilelik kontrolleri size, çocuğun ana karnındaki gelişimini izlemenizde yardımcı olacaktır ama, anne olarak, doğumdan önce çocuğu en iyi siz tanıma imkanına sahipsiniz. Çocuğun, tekmeleme yoluyla anlatmak istediği şeyler, önemlidir!

Doğum sonrası çocuğunuzla ilgilenmeye çok zaman harcayacaksınız. Biz size, hamileliğiniz sırasında her gün, çocuğun hareketlerini hissetmek yoluyla onunla tanışmaya biraz zaman ayırmanızı tavsiye ederiz. Burada, çocuk tekmelediğinde bunun ne anlama geldiğine ilişkin bilgi ve çocuğun hayatından memnun olup olmadığını nasıl izleyebileceğiniz konusunda tavsiyeler bulacaksınız.

Çocuğunuzla tanışmaya zaman ayırmanız iyi bir alışkanlık olur!

Tekmeleme, çocuğun hoşnutluğu açısından ne anlama geliyor?

Çocuğun ihtiyacı olan her şey plasenta yoluyla sizden ona nakledilir. Bu tedarik işlemi iyi gittiği sürece, çocuk tekmeler. Plasentadan nakledilen tedarik az olduğunda, veya çocuk hastalandığında, büyümeye devam edebilmek için gücünü idareli kullanmak zorundadır – böylece tekmeleme azalır. Sigara içiyorsanız, hem plasenta ve hem de çocuk etkilenecek ve tekmeleme azalacaktır. Plasentanın işlevinin ciddi bir şekilde zayıflaması halinde, bu çocuğun büyümesini engeller ve çocuk ciddi olarak hastalanabilir veya zarar görebilir. Böylece doğuma dayanıklılığı da zayıflayabilir. Çocuğun normal olarak tekmelemesi durumunda bu ihtimal çok seyrek olarak ortaya çıkar!

Bazen çocuk sessiz sedasızdır. Burada dikkat edilecek nokta, çocuğun olağan tekmelesinin çok azalması durumudur. Karnınızdaki olağan hayat ritmini hissettiğiniz sürece, bu çocuğun durumunun iyi olduğuna işaret eder. Çocuğun hareketlerini hissetme çabanız, çocuğunuzla aranızda daha yakın bir bağ kurulmasına katkıda bulunur. O nedenle, çocuğun tekmelemesini hesaba katmak doğru bir davranıştır.

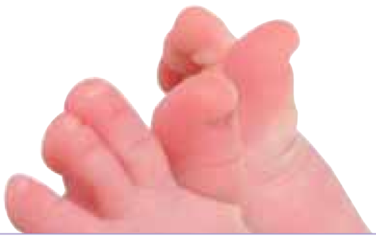

O nedenle çocuğun tekmelemesini hesaba katarız!

Karnınızda tekmeleme hissettiğinizde – bu ne anlama gelir?

Çocuk karnınızda neler yapıyor?

Çocuğunuz tüm hamilelik boyunca hareket halinde olacaktır. Onun en çok yaptığı, sizin hissetmediğiniz, soluk alıp verme hareketleridir. Doğum sonrası için alıştırmalar yaparken, böylece akciğerleri genişlemektedir. Çocuğu ara sıra hıçkırık tutar, siz bunu düzenli küçük irkilmeler olarak hissedersiniz.

Çocuk karnınızda, hem büyük, hem de küçük hareketler yapar. Elle küçük kavrama hareketlerini, baş parmağını emmesini veya el ve ayak parmaklarını kapayıp açmasını pek hissetmezsiniz. Bunun yanısıra, hamileliğin son döneminde, çocuğun karnınızda tekme ve itme gibi hareketlerinin pek çoğunu gayet iyi hissedersiniz. Örneğin siz ayakta durur durumdan yatar duruma geçtiğinizde ortaya çıkan daha büyük beden hareketleri de genellikle açık seçik hissedilir. Bu durumda çocuk da biraz dönmekte, sizin onu hafifçe ittiğinizde onun da genellikle iterek karşılık verdiği durumlarda olduğu gibi, yer değiştirmektedir.

Bazen sessiz sedasız....

Gün boyunca çocuğunuz pek çok kez uyur ve o anlarda tamamen sessizdir. Hamilelik ilerledikçe uyku süreleri de uzar, ama tahmini doğum tarihi yaklaştığında bile çocuk seyrek olarak her keresinde bir saatten fazla uyur. Çocukların çoğu en çok akşamları, ve bir çoğu da sabah erken saatlerde hareket halindedir.

Ne kadar yoğun ve güçlü ”tekmeledikleri” konusu çocuktan çocuğa büyük farklılık gösterebilir – burada, tekmeleme ile her türlü hareketi kastediyoruz. Çok tekmeleyen çocuk genellikle doğum sonrası da daha hareketli olur. Bu, çocuğun sağlığı açısından bir gösterge değildir. Bu konuda kız çocuklarla erkek çocuklar arasında bilinen bir farklılık yoktur.

Bazı anneler, tekmelemeleri diğer annelere göre daha az hisseder. Eğer plasenta rahmin ön yanında yer alıyorsa veya fazla kiloluysanız, daha az hissedersiniz. Karnınızın hareket edip etmediğine bakarken aynı zamanda tekmelemeyi hissetmeye çalışarak kendi kendinize deneme yapabilirsiniz. Tekmelemeyi en iyi yatar durumdayken, en az da ayakta dururken, yürürken veya bir şeylerle meşgul olurken hissedersiniz.

Fotoğraf: Per Oscar Skjellnan
Çizim: Marianne Bratt Ricketts • www.mariannedesign.no

Sorunuz var mı? Bakınız, www.telltrivselen.no

Tekmelemeyi hesaba katmak ne anlama geliyor?

Çocuğun hareketlerini her gün hissetmeye çalışmak iyi bir alışkanlıktır, ve tekme sayma şeması, çocuğun tekmelemesi konusunda size ve ebeye/doktora fikir veren basit bir yardımcı malzemedir. Bu, çocuğunuz için nelerin normal olduğunu daha kolay görmenizi sağlar. Tekmeleme herkes için önemli olsa da, eğer ikiz çocuklara hamile değilseniz, tekme sayma şeması kullanıma, en çok hamileliğin 28. haftasından sonra elverişlidir. Tekme sayma şemasını doldurup doğumdan sonra iade edecek olursanız, tekmeleme faaliyetinden yola çıkarak, hamilelikte ekstra yardıma ihtiyaç olup olmadığının nasıl anlaşılabileceği konusunda yapılan önemli araştırmalara da katkıda bulunmuş olursunuz. Böyle bir araştırmaya katılmak isterseniz, bu konudaki bilgileri dosyanın arka yüzünde bulabilirsiniz.

Nasıl hesap edilir?

Hamileliğin 28. haftasına geldiğinizde tekmeleri hesap etmeye başlayın. Başladığınız tarihi ve size verilmiş olan tahmini doğum tarihini şemaya yazın.

10 tekme sayın. İlk tekmeyi hissettiğiniz andan itibaren saat tutun (böylece çocuğun uyanık olduğunu bilirsiniz). Bütün hareketler “tekmeleme” sayılır, fakat hıçkırıkları hesaba katmayacaksınız! Aynı anda meydana gelen bir çok hareket, bir “tekme” sayılır. Bunu en çabuk, yatar/rahat oturur vaziyette ve hareketleri iyice hissetmeye çalışarak yapabilirsiniz. Eğer çocuk uyuyorsa, onu, karnınızı okşayarak/iterek veya soğuk bir şeyler içerek uyandırmanın bir sakıncası yoktur.

Şemada, 10 tekme sayana kadar geçen zaman ve hangi gün olduğunu gösteren haneye çarpı işareti koyun.

Mümkün olduğunca, her gün yaklaşık aynı zamanda saymaya başlayın. Gün içinde, biraz zaman ayırabileceğiniz, size en uygun ve çocuğun hareketli olduğu anı seçin – tercihen sabah yataktan kalkmadan önce. Sayıma her gün aynı iki saat içinde başlayın. Genel olarak hangi saatlerde saydığınızı şemaya yazın.
Böyle yapılacak olursa, pek çok anne büyük olasılıkla 15 dakika içinde tekme sayma işlemini bitirecektir.

__Tekme sayma şeması nasıl doldurulacak__:

Şemadaki haneyi mavi/siyah tükenmez kalemle işaretleyin. 10 tekme sayana kadar ne kadar zaman geçtiğini belirtin.

__Örnek:__
Eğer 10 tekme sayana kadar 30 dakika geçtiyse, 26-30 dakika hanesine çarpı işareti koyun.

Eğer 10 tekme sayana kadar 1 saat ve 5 dakika geçtiyse, 2. saatin altındaki 0-5 dakika hanesine çarpı işareti koyun.

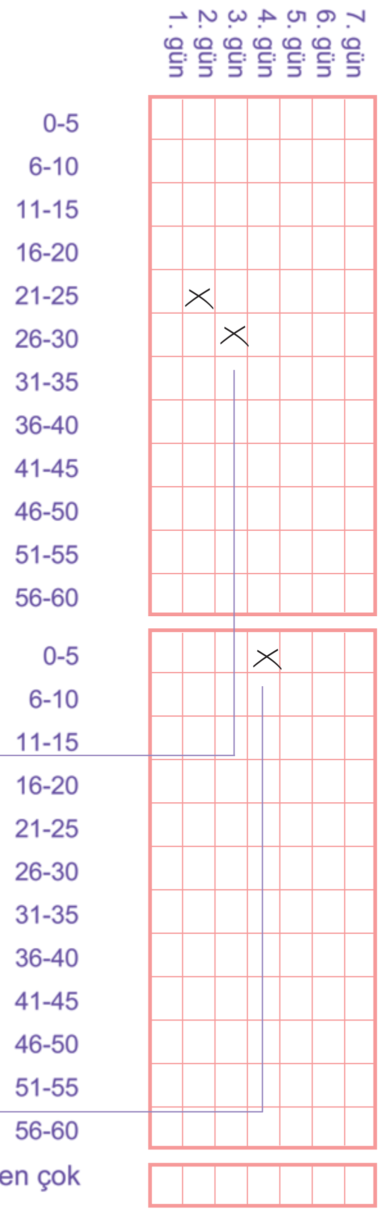

Bebek ne kadar tekmelemeli – ve tekmeleme azalırsa ne olur?

Tekme sayma şemasını bir veya iki hafta kullandıktan sonra, tekmelemenin günden güne biraz farklılık gösterdiğini ama, günlerin büyük oranda birbirine benzediğini göreceksiniz. Hamilelik sırasında çocuğun hareketlerini hissedişinizde değişiklik meydana gelse bile, halinden memnun olan bir çocuk için durum bu şekilde gelişecektir. En önemlisi, çocuğunuzun faaliyetlerinde, normal faaliyetlerine kıyasla, büyük ve sürekli bir düşüş olmamasıdır! Tekme sayma şeması, bunu gözlemekte size yardımcı olur.

Çocuğunuz için endişeleniyorsanız, nedeni ne olursa olsun, yardım ve tavsiye almak için başvurmanızda yarar vardır. Çocuğunuzun haftalar ilerledikçe giderek daha az tekmelemesi sizi endişelendiriyorsa, bir sonraki hamilelik kontrolüne giderken tekme sayma şemasını yanınıza alın.

Bazı durumlarda direk olarak hastahanenin doğum bölümü ile temasa geçmelisiniz:

- *Eğer çocuğunuz bir gün tekmelemiyorsa, **kesinlikle** bir sonraki güne kadar beklemeyin.*
- *Eğer çocuk bir gün/günler içinde giderek daha az tekmeliyorsa, ve siz karnınızda ”az canlılık” hissediyorsanız.*

”Az canlılık” ile ne kastedildiğinden emin değilseniz, sağlıklı bir çocuğun, genellikle faal olduğu iki saat içinde, en az 10 kez tekmelediğini bilmenizde yarar var. Gün boyunca çocuğun çok az tekmelediğini hissettiyseniz, hastahanenin doğum bölümü ile temasa geçmelisiniz. Eğer çocuğun günlük tekmelerini saymaya başlamadan önce de çok az tekmeleyip etmediğini bilmiyorsanız, durumu izlemeniz gerekir. Sonraki 12 saat içinde sayımı tekrarlayın ve sonuç değişmiyorsa hastahanenin doğum bölümü ile temasa geçin.

Not: Haneyi tükenmez kalemle işaretleyin

Bugün saymaya başladım (tarih, gün.ay.yıl) ☐☐.☐☐.☐☐

Tahmini doğum yapma zamanım (tarih, gün.ay.yıl) ☐☐.☐☐.☐☐

Genellikle şu saatler arasında sayıyorum saat: ☐☐.☐☐ ve saat: ☐☐.☐☐

HAFTANIN GÜNLERİ

SAYMAYA BAŞLAYIN

DAKİKALAR

İlk saat – her hane için 5 dakika

2. saat – her hane için 5 dakika

	1. gün	2. gün	3. gün	4. gün	5. gün	6. gün	7. gün	1. gün	2. gün	3. gün	4. gün	5. gün	6. gün	7. gün	1. gün	2. gün	3. gün	4. gün	5. gün	6. gün	7. gün	1. gün	2. gün	3. gün	4. gün	5. gün	6. gün	7. gün	1. gün	2. gün	3. gün	4. gün	5. gün	6. gün	7. gün	1. gün	2. gün	3. gün	4. gün	5. gün	6. gün	7. gün	1. gün	2. gün	3. gün	4. gün	5. gün	6. gün	7. gün
0-5																																																	
6-10																																																	
11-15																																																	
16-20																																																	
21-25																																																	
26-30																																																	
31-35																																																	
36-40																																																	
41-45																																																	
46-50																																																	
51-55																																																	
56-60																																																	
0-5																																																	
6-10																																																	
11-15																																																	
16-20																																																	
21-25																																																	
26-30																																																	
31-35																																																	
36-40																																																	
41-45																																																	
46-50																																																	
51-55																																																	
56-60																																																	
Iki saatten çok																																																	
	28. HAFTA							29. HAFTA							30. HAFTA							31. HAFTA							32. HAFTA							33. HAFTA							34. HAFTA						

HAFTANIN GÜNLERI — DAKIKALAR

42. HAFTA

HAFTANIN GÜNLERI	İlk saat 0-5	6-10	11-15	16-20	21-25	26-30	31-35	36-40	41-45	46-50	51-55	56-60	2. saat 0-5	6-10	11-15	16-20	21-25	26-30	31-35	36-40	41-45	46-50	51-55	56-60	İki saatten çok
7. gün																									
6. gün																									
5. gün																									
4. gün																									
3. gün																									
2. gün																									
1. gün																									

41. HAFTA

HAFTANIN GÜNLERI	İlk saat 0-5	6-10	11-15	16-20	21-25	26-30	31-35	36-40	41-45	46-50	51-55	56-60	2. saat 0-5	6-10	11-15	16-20	21-25	26-30	31-35	36-40	41-45	46-50	51-55	56-60	İki saatten çok
7. gün																									
6. gün																									
5. gün																									
4. gün																									
3. gün																									
2. gün																									
1. gün																									

40. HAFTA

HAFTANIN GÜNLERI	İlk saat 0-5	6-10	11-15	16-20	21-25	26-30	31-35	36-40	41-45	46-50	51-55	56-60	2. saat 0-5	6-10	11-15	16-20	21-25	26-30	31-35	36-40	41-45	46-50	51-55	56-60	İki saatten çok
7. gün																									
6. gün																									
5. gün																									
4. gün																									
3. gün																									
2. gün																									
1. gün																									

39. HAFTA

HAFTANIN GÜNLERI	İlk saat 0-5	6-10	11-15	16-20	21-25	26-30	31-35	36-40	41-45	46-50	51-55	56-60	2. saat 0-5	6-10	11-15	16-20	21-25	26-30	31-35	36-40	41-45	46-50	51-55	56-60	İki saatten çok
7. gün																									
6. gün																									
5. gün																									
4. gün																									
3. gün																									
2. gün																									
1. gün																									

38. HAFTA

HAFTANIN GÜNLERI	İlk saat 0-5	6-10	11-15	16-20	21-25	26-30	31-35	36-40	41-45	46-50	51-55	56-60	2. saat 0-5	6-10	11-15	16-20	21-25	26-30	31-35	36-40	41-45	46-50	51-55	56-60	İki saatten çok
7. gün																									
6. gün																									
5. gün																									
4. gün																									
3. gün																									
2. gün																									
1. gün																									

37. HAFTA

HAFTANIN GÜNLERI	İlk saat 0-5	6-10	11-15	16-20	21-25	26-30	31-35	36-40	41-45	46-50	51-55	56-60	2. saat 0-5	6-10	11-15	16-20	21-25	26-30	31-35	36-40	41-45	46-50	51-55	56-60	İki saatten çok
7. gün																									
6. gün																									
5. gün																									
4. gün																									
3. gün																									
2. gün																									
1. gün																									

36. HAFTA

HAFTANIN GÜNLERI	İlk saat 0-5	6-10	11-15	16-20	21-25	26-30	31-35	36-40	41-45	46-50	51-55	56-60	2. saat 0-5	6-10	11-15	16-20	21-25	26-30	31-35	36-40	41-45	46-50	51-55	56-60	İki saatten çok
7. gün																									
6. gün																									
5. gün																									
4. gün																									
3. gün																									
2. gün																									
1. gün																									

35. HAFTA

HAFTANIN GÜNLERI	İlk saat 0-5	6-10	11-15	16-20	21-25	26-30	31-35	36-40	41-45	46-50	51-55	56-60	2. saat 0-5	6-10	11-15	16-20	21-25	26-30	31-35	36-40	41-45	46-50	51-55	56-60	İki saatten çok
7. gün																									
6. gün																									
5. gün																									
4. gün																									
3. gün																									
2. gün																									
1. gün																									

İlk saat — 2. saat — İki saatten çok

Not: Haneyi tükenmez kalemle işaretleyin

Şemayı doldurduğunuzda şu adrese gönderin veya teslim edin:

Tekmeleme faaliyeti ve sağlık arasındaki bağlantıyla ilgili araştırmaya katılmak ister misiniz?

Çocuk doğurmanız halinde, çocuğunuzu doğurduktan sonra, **bu dosyayı iade ederek** araştırmaya katılmanızı arzu ediyoruz. Tekme sayma şemasını kullanmış olsanız da olmasanız da araştırmaya katılabilirsiniz.

Kim ve ne için?
Şimdilerde, Folkehelseinstituttet (Halk Sağlığı Enstitüsü), Perinatalmedisinsk forskningssenter, kvinneklinikken Rikshospitalet-Radiumhospitalet HF (Devlet Hastahanesi Doğum Süreci Tıbbi Araştırma Merkezi, Kadın Kliniği-Radyum Hastahanesi) ve Sanitetskvinnene (Sıhhiye Kadınları) yönetiminde çocuğun ana karnındaki tekmeleme faaliyetleri ve gelecekteki sağlık durumu arasındaki bağlantıyla ilgili araştırmalar yapılmaktadır. Tekmeleme faaliyetinde düşüş veya bu faaliyetin kesilmesinin hastalık ve ölüm tehlikesi yarattığını biliyoruz ama, bu ve çocuğun ana karnındaki tekmeleme faaliyetiyle ilgili diğer bilgilerin, çocuğun kısa ve uzun vadede sağlık durumunun korunmasında nasıl kullanılabileceği konusundaki bilgilerimiz yeterli değil.

Araştırmaya katılım tamamen gönüllüdür, az veya çok katkıda bulunabilirsiniz.
Araştırma açısından, mümkün olduğunca çok sayıda kişinin, doğum sonrası, doğum/loğusa bölümünden ayrılmadan önce, bu dosyayı iade etmeleri önemlidir. Tekmeleme şemasını kullanmamış olsanız veya kendiniz hakkında bilgi vermek istemiyor olsanız bile, bu dosya, araştırma açısından değer arzetmektedir. Ancak bu değer siz katkı sağladıkça artar. Eğer 18 yaşından küçükseniz, velilerinize danışmanız gerekir. Ne derece katkıda bulunacağınızı şöyle seçebilirsiniz:

Anonim kalmak ister misiniz?
O takdirde bu yazının sonundaki bilgileri doldurun ve buraya çarpı işareti koyarak **onay** verdiğinizi belirtin: ☐

Daha fazla katkıda bulunabilir misiniz? Doğum tarihi ve kimlik numaranızı belirterek ve burayı imzalayarak, dosyanızdaki bilgilerin Tıbbi Doğum Kayıtlarına eklenmesine **onay** verebilirsiniz:
İmza:________________________________ kimlik numarası ☐☐ ☐☐ ☐☐ ☐☐☐☐☐

Veya
Eğer Norveçli anne ve çocuk araştırmasına katılıyorsanız, kimlik numaranızı belirterek ve burayı imzalayarak, dosyanızdaki bilgilerin araştırmaya eklenmesine **onay** verebilirsiniz:
İmza:________________________________ kimlik numarası ☐☐ ☐☐ ☐☐ ☐☐☐☐☐

Araştırmaya katılım güvenli midir?
Halk Sağlığı Enstitüsü, verdiğiniz bilgilerin gizli kalması ve güvenliğinden sorumludur. Enstitü, sizinle ve çocuğunuzla ilgili bilgilerin korunması konusunda Bilgisayar Kayıt Denetmenliğinden (Datatilsynet) ruhsat ve Araştırma Etiği Komitesinden (Forskningsetisk komité) tavsiye almıştır.
Halk Sağlığı Enstitüsü, hakkınızdaki bilgilerinizi kaydettikten ve onay verdiğiniz bilgilerin size ait olduğunu belirttikten hemen sonra, sizinle ilgili bilgileri anonim hale getirecektir. Bu, dosyanızın yok edilmesi ve araştırmacılar görmeden önce, hakkınızdaki tüm kimlik belirten bilgilerin ortadan kaldırılması anlamına gelmektedir.

Dosyayı nereye vereceğim?
Tüm doğum ve loğusa bölümlerinde kolaylıkla görülen kutular mevcuttur. Emin olamazsanız personele sorun.

Herhangi bir bilgi vermek istemiyorum................................ ☐

Hamileliğin kaçıncı haftasında doğum yaptınız? .... ☐☐
(40. hafta tahmini doğum tarihidir)

Nasıl doğum yaptınız?
Normal doğum (döl yoluyla).. ... ☐
Önceden planlanmış sezaryen... ☐
Doğum sırasında karar verilen sezaryen........................... ☐

Acil sezaryen.. ☐
Çocuğun cinsiyeti Oğlan ☐ Kız ☐
Çocuğun doğum kilosu ☐☐☐☐ gram

Çocuğun durumu nasıl gitti?
Çocuğun sağlığı yerindeydi... ☐
Çocuk, yeni doğanlar bölümüne/çocuk bölümüne yatırıldı ☐
Çocuk vefat etti.. ☐

Sizin yaşınız.. ☐☐ yaş

Hamilelik öncesi kilonuz............................ ☐☐☐ kg olarak

Boyunuz.. ☐☐☐ cm olarak

Daha önce kaç çocuk doğurdunuz............................... ☐☐

Bu hamileliğinizin son ayında sigara içiyor muydunuz?
Hayır....... ☐
Ara sıra.... ☐ ☐☐ sigara, **haftada**
Günlük..... ☐ ☐☐ sigara, **günde**

Norveççe dışında anadiliniz var mı? EVET ☐ HAYIR ☐

Cevap EVET ise, hangi dil? ☐☐☐☐☐☐☐☐☐☐

Yardımlarınız için teşekkürler!

folkehelseinstituttet

Rikshospitalet – Radiumhospitalet HF

Oversatt ved Tolketjenesten i Oslo.
November 2005

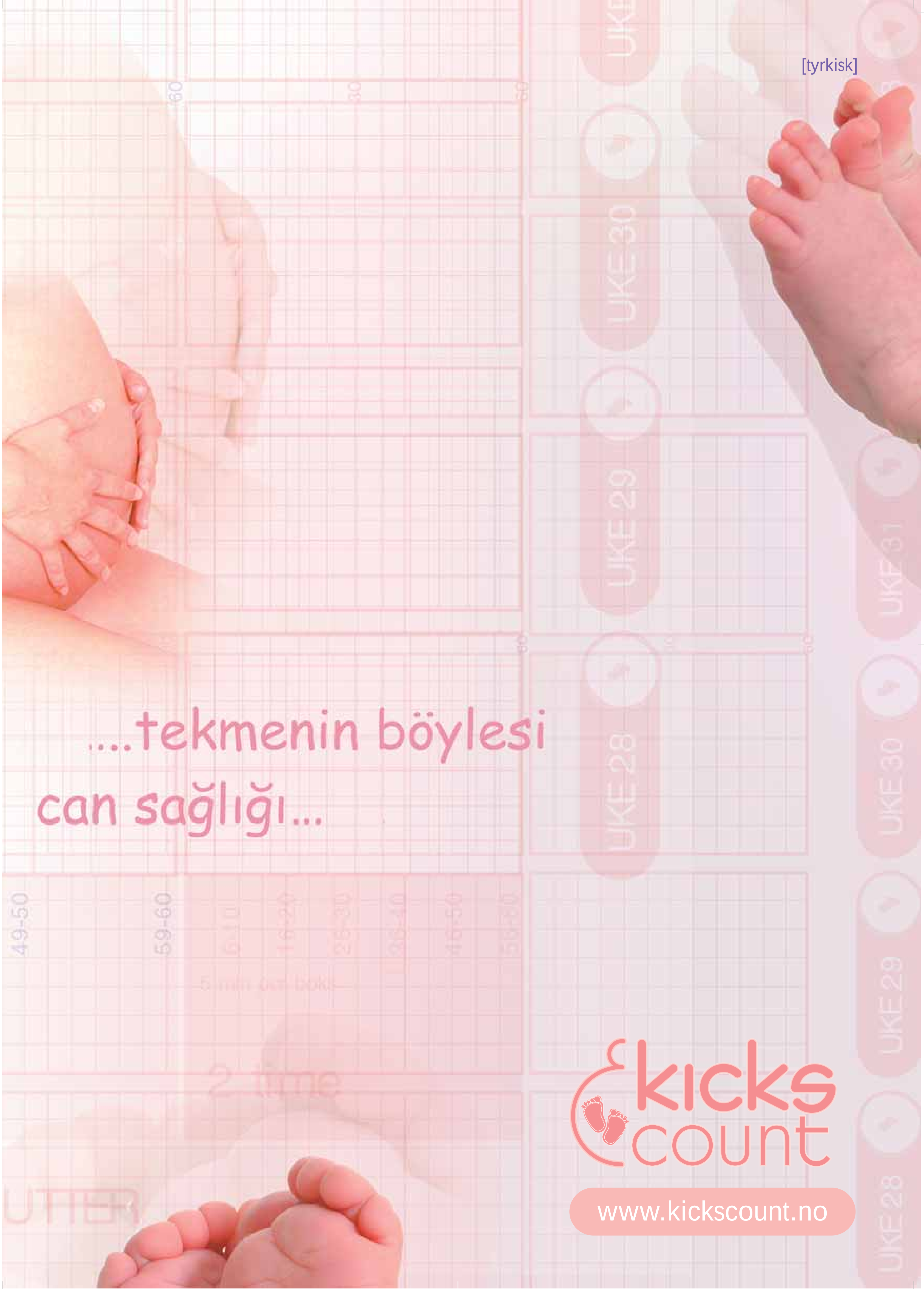
[tyrkisk]
....tekmenin böylesi
can sağlığı...
www.kickscount.no
