# Supplementary material for: Reduction of late stillbirth with the introduction of fetal movement information and guidelines – a clinical quality improvement
Source: BMC Pregnancy Childbirth. 2009 Jul 22;9:32. doi: 10.1186/1471-2393-9-32 (PMC2734741; doi:10.1186/1471-2393-9-32)
Supplement: Additional file 5 — Kicks Count. Kicks Count brochure, Somali version. A brochure of information aiming to increase maternal awareness and vigilance to significant decreases in fetal activity, and to aid health promoting behavior. The brochure was provided as a part of the routine information given to women at the standard ultrasound assessment at 17–19 weeks in Norway as a part of the quality improvement intervention. [file 1471-2393-9-32-S5.pdf]

ISBARTA ILMAHAAGA!

Hadda uur waxoogaa ah ayaad leedahay waxana laga yaabaa in aad dareemayso ilmaha gujadiisa. Shaqaalaha caafimaadka ee ku caawiya muddada uurka ayaa kugu caawin doona sida aad ula soconayso xaaladda ilmaha, laakiin adiga oo hooyadii ah ayaa noqon kara qofka sida ugu fiican uga war haya xaaladda ilmaha inta aanu dhalan. Waxa uu ilmuhu ku muujinayo gujadu waa muhiim.

Umusha ka dib waxa aad wakhti badan ku isticmaali doontaa sidii aad ilmaha wax ugu qaban lahayd. Waxa aanu kugula talinaynaa in aad inta aad uurka leedahay maalin walba wakhti yar ku isticmaasho sidii aad u baran lahayd ilmaha adiga oo baranaya dhaqdhaqaaqiisa. Halkan waxa aad ka heli kartaa warbixin iyo talooyin ku saabsan maxay xambaarsan tahay gujada ilmuhu iyo sidee ula socon kartaa in ilmuhu ku qanacsan yahay xaaladdiisa.

Waa caado wanaagsan in wakhti la siiyo barashada xaaladda ilmaha!

MAXAY GUJADU KA SHEEGAYSAA KU QANACSANAANTA ILMAHA EE XAALADDIISA?

Wax kasta oo ilmuhu u baahan yahay waxa ay u soo maraan mandheerta. Inta ay nafaqadu ilmaha si fiican ugu socoto gujadiisuna way socon. Haddii ay yaraato nafaqada soo gaadhaysaa ama uu ilmuhu xanuunsado, waxa markaa dhacaysa in uu dhaqaalaysto awoodda si uu koritaankiisu u sii socdo, waxana uu markaa yareeyaa gujadii. Haddii aad sigaarka cabto, waxa uu saamayn ku samaynayaa mandheerta iyo ilmaha labadaba, isla markaana gujadii way yaraanaysaa. Haddii dhaawaca gaadhay mandheertu uu halis yahay, waxa istaagaya koritaankii ilmaha, isla markaana ilmuhu xoog ayuu u xanuunsanayaa ama dhaawac ayaa soo gaadhaya. Waxa sidoo kale markaa dhici karta in uu xamili waayo soo dhalashada. Arrintan waxa ay dhacdaa marmar iyo dhif haddii gujada ilmuhu ay caadi soo ahayd.

Marmarka qaar ilmuhu wuu iska deggan yahay iyada oo aanay dhibi jirin. Arrinta muhuumka ahi waa haddii gujadii ilmaha ee caadiga ahayd in badan la waayo. Inta aad dareensan tahay dhaqdhaqaaqa caadiga ah ee nolosha ilmaha calooshaada ku jira, waxa ay muujinaysaa in ilmuhu fiican yahay. In aad dareento gujooyinka ilmuhu, waxay ka qayb qaadanaysaa sida aad xidhiidh ula yeelanayso ilmahaaga aad uurka ku siddo. Sababtaas awgeed ayay u muhiim tahay in aad tiriso gujada ilmaha oo markhaati u ah in uu ku qanacsan yahay xaaladdiisa!

Sidaa darteed ayaynu u tirinaa farxad muujinta.

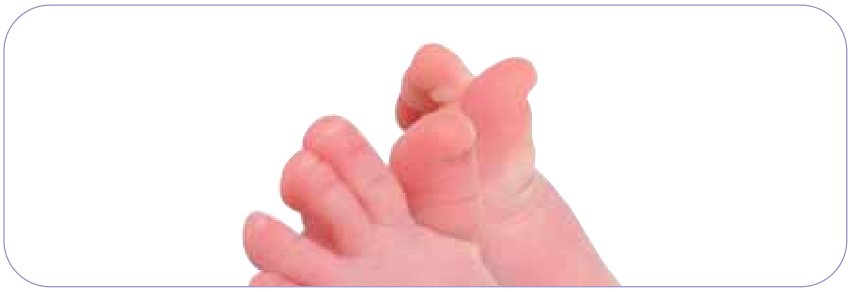

Marka ilmuhu caloosha kaa gujiyo - waa maxay macnaheedu?

MUXUU ILMUHU KA QABTAA CALOOSHA DHEXDEEDA?

Ilmahaagu waxa uu noqonayaa mid firfircoon muddada uurka oo idil. Waxa ugu badan ee uu caloosha dhexdeeda ka sameeyaa waa dhaqdhaqaaq neefsasho oo aanad adigu dareemayn. Sidaa ayayna sambabadu ku kala durkaan oo ay ugu diyaar garoobaan wakhtiga dhalmada ka dib. Marmarka qaar ayuu ilmuhu higgoodaa oo aad dareentaa bootimo yaryar oo si nadaamsan u soo baxaya.

Ilmuhu waxa uu calooshaada dhexdeeda ka samaynayaa dhaqdhaqaaqyo waaweyn iyo kuwa yaryar labadaba. Dhaqdhaqaaqyo yaryar, suul nuugid ama kala bixin faraha gacmaha iyo lugaha oo uu ilmuhu sameeyo ayaa laga yaabaa in aanad adigu dareemin. Laakiin inta ugu badan ee gujooyinka iyo riixniinka uu ilmuhu caloosha ka dhex sameeyo waad dareentaa gaar ahaan marxaladaha ugu dambeeya ee uurka. Waxa kale oo inta badan muuqda dhaqdhaqaaqyada jidheed ee waaweyn oo iman kara tusaale ahaan marka aad iska bedesho xaalad ee aad jiifsato adiga oo taagnaa. Markaa ilmuhu wuu is yara rogaa, wuuna yara dhaqdhaqaaqaa isaga oo ku soo caabinaya marka aad yara riixdo.

MARMAR WAA BILAA XARAKO...

Marar badan ayuu maalintii ilmuhu hurdaa oo markaa aanad dareemayn wax xarako ah haba yaraatee. Inta ilmuhu hurdayaa way dheeraataa marba marka ka dambaysa, laakiin marka wakhtiga umushu soo dhowaado dhif iyo naadir ayuu ilmuhu seexdaa wax ka badan saacad markiiba. Carruurta uurka ku jirta inta ugu badani waxa ay firfircoon yihiin galabtii, qaar badanina sidoo kale waxa ay firfircoon yihiin subaxa hore.

Carruurtu aad ayay ugu kala duwan tahay dhinaca inta goor ee ay sameeyaan gujo, iyo sida u xoog badan karto gujadu. Gujada halkan waxa looga jeedaa dhammaan noocyada dhaqdhaqaaq ee ilmuhu sameeyo. Ilmaha gujada badan leh uurku, waxa uu noqdaa mid firfircooni badan dhalashada ka dibna. Laakiin kan gujada badan iyo ka degganiba waxa ay labaduba noqon karaan kuwo isku si u caafimaad qaba. Lama oga in ay kala duwan yihiin wiilasha iyo gabdhuhu dhinaca gujada.

Hooyooyinka qaar ayaa dareenkooda gujooyinku uu ka liitaa kuwa kale. Haddii mandheertu ay ku jirto dhinaca hore ee ilmagaleenka ama uu adiga miisaankaagu weyn yahay, waxa aad dareemaysaa gujooyin ka yar kuwa hooyooyinka kale. Waxa aad ku tababaran kartaa sida feejignaan loogu yeesho gujada isla markaana adiga oo eegaya in calooshu dhaqdhaqaaqayso. Gujooyinka waxa aad sida ugu fiican u dareemi kartaa marka aad jiifto, inta ugu yarna marka aad taagan tahay, soconayso ama aad wax ku mashquulsan tahay.

Sawir: Per Oscar Skjellnan • www.peroscar.no

Qaab: Marianne Bratt Ricketts • www.mariannedesign.no

Su'aal ma qabtaa ? Eeg bogga internett ka: www.telltrivselen.no

TIRINTA FARXAD MUUJINTA?

In la dareemo gujo maalin walba waa caado wanaagsan, foomka gujaduna waa caawiye fudud oo sawir ka siinaya adiga iyo umulisadaada/ dhakhtarkaaga dhaqdhaqaaqa ilmaha. Arrintani waxa ay fududaynaysaa in la arko in xaaladda ilmahaagu tahay caadi. Inkasta oo ay gujadu u fiican tahay dhammaan, haddana foomka gujadu waxa uu ku sii fiican yahay marka laga gudbo toddobaadka 28.aad ee uurka, iyo haddii aanad mataano sidin. Haddii aad soo buuxiso foomka gujada oo aad dhiibto umulidda ka dib, waxa aad caawimo ka gaysanaysaa cilmibaadhis muhiim ah oo ku saabsan sidii loo ogaan lahaa xaaladda u baahan caawimo dheeraad ah iyada oo laga tixraacayo dhaqdhaqaaqa gujada. Haddii aad doonayso in aad ka qayb qaadato cilmibaadhista sidan ah, waxa aad ka helin kartaa warbixin arrintan ku saabsan dhinaca dambe ee galka.

SIDEE TIRINTA LOO SAMAYNAYAA?

Bilow tirinta farxad muujinta marka la soo gaadho toddobaadka 28.aad ee uurka. Foomka ku qor taariikhda aad bilaabayso iyo wakhtiga umushu ku beegan tahay.

Tiri ilaa 10 – qabo wakhtiga oo ka bilow marka aad dareento gujada ugu horraysa (markaa waxa aad og tahay in ilmuhu soo jeedo). Dhammaan dhaqdhaqaaqyada waxa aad ka soo qaadaysaa in ay yihiin ”gujo”, laakiin higgada kuma tirinaysid. Dhaqdhaqaaqyada dhowrka ah ee hal mar wada dhaca waxad ka dhigaysaa hal gujo. Waxa ay ugu fiican tahay marka aad jiifto/ ama u fadhido si deggan oo aad si fiican u dhegaysanayso. Haddii ilmuhu hurdo, waa la ogol yahay in aad kiciso adiga oo isticmaalaya sacab yar/caloosha oo aad riixdo ama adiga oo caba wax qabow.

Isku tallaab ku muuji halka loogu talagalay ee foomka wakhtiga ay qaadatay in aad tiriso 10 gujo iyo taariikhda maalintaas.

Maalin walba xilli isku mid ah tirada samee – sida ugu badan ee suuragal ah. Ka dooro wakhtiga kugu habboon 24 ka saacadood si aad wakhti yar u siisid arrintan weliba marka aad og tahay in ilmuhu soo jeedo oo firfircoon yahay- waxa ugu fiican subaxa hore inta aanad kicin. Tirinta bilow maalin walba laba saacadood oo isla wakhtigii ku beegan gudahood. Foomka ku qor wakhiga aad caadaysatay in aad tiriso gujada.

Haddii aad sidaa samayso, waxa ay inta ugu badani ku isticmaalaan tirinta farxad muujinta in ka yar 15 daqiiqo.

Gujo intee le'eg ah ayay tahay in ilmuhu sameeyo - maxaase dhacaya haddii uu joojiyo?

Marka aad isticmaasho foomka gujada hal ama laba toddobaad ayaad arki kartaa in gujadu ay waxoogaa kala duduwan tahay oo maalinba si tahay, laakiin ay maalmuhu guud ahaan isku dhowdhow yihiin. Sidaa ayay u soconaysaa hawsha ilmaha ku qanacsan xaaladda uu jiraa in kastoo habka aad u dareemayso dhaqdhaqaaqu uu is bedbedelayo inta aad uurka leedahay. Waxa ugu muhiimsani waa in aanad firfircoonida caadiga ah ee ilmaha ka dareemin hoos u dhac weyn oo joogto ah. Foomka gujada ayaa kugu caawinaya in aad aragto xaaladdan.

Haddii aad u werwersan tahay ilmahaaga, sababtu waxa ay doonto ha ahaatee, waxa kula gudboon in aad raadsato caawimo iyo talobixin. Haddii sababta werwerku tahay in ilmahaagii uu si tartiib ah u soo yaraynayo dhaqdhaqaaqii, wakhtigiina uu socdo, waxa habboon in aad soo qaaddo foomka gujada maalinta aad ballanta hubinta caafimaadkaaga iyo ka ilmaha leedahay.

Xaaladaha qaar ayay habboon tahay in aad toos ula soo xidhiidho qaybta dhalmada ee dhakhtarka:

- Haddii aad ilmaha ka weydo wax dhaqdhaqaaq ah maalin dhan, weligaa ha sugin maalinta xigta.
- Haddii dhaqdhaqaaqa ilmuhu sii yaraado maalin/ maalmo, oo aad dareento ”dhaqdhaqaaq yaraan”.

Sida loo buuxiyo foomka tirinta gujada:

Godkan isku tallaab ku samee qalin buluug/ madow ah. Sheeg wakhti intee ah ayay qaadatay tirinta 10 gujo.

Tusaale:
Haddii ay qaadatay 30 daqiiqo tirinta 10 gujo, iskutallaab ku samee godka 26-30 daqiiqo.

Haddii ay qaadatay 1 saac iyo 5 daqiiqo tirinta 10 gujo, waxa aad isku tallaab ku samaynaysaa godka 0-5 daqiiqo ee saacadda 2.aad.

		Maalinta 1.aad	Maalinta 2.aad	Maalinta 3.aad	Maalinta 4.aad	Maalinta 5.aad	Maalinta 6.aad	Maalinta 7.aad
Saacadda 1.aad	0-5							
	6-10							
	11-15							
	16-20							
	21-25	X						
	26-30		X					
	31-35							
	36-40							
	41-45							
	46-50							
	51-55							
	56-60							
Saacadda 2.aad	0-5				X			
	6-10							
	11-15							
	16-20							
	21-25							
	26-30							
	31-35							
	36-40							
	41-45							
	46-50							
	51-55							
	56-60							
In ka badan 2 saacadood.								

Daqiiqado

Haddii aad is weydiiso maxaa ah ”dhaqdhaqaaq yari”, waxa habboon in aad ogaato in ilmo caafimaad qabaa ay dhif tahay in uu dhaqdhaqaaqo oo gujo sameeyo in ka yar 10 goor laba saacadood gudahood oo aad og tahay in ay tahay wakhtigii uu firfircoonaan jiray. Haddii aad dareento in dhaqdhaqaaqiisu yaraa maalintii oo dhan, waxa habboon in aad la soo xidhiidho qaybta dhalmada ee dhakhtarka. Haddii aanad kala ogayn in dhaqdhaqaaqiisu yaraa intii ka horraysay marka aad bilowday tirinta dhaqdhaqaaqa maalintaa, waxa fiican in aad si fiican ula socoto xaaladdiisa. Isku day markaa in aad dhaqdhaqaaqiisa tiriso mar kale 12 ka saacadood ee soo socda oo la xidhiidh qaybta dhalmada ee dhakhtarka haddii arrintu is bedeli weydo.

F/G: Iskutallaabta godka waxa aad ku samaynaysaa qalin biiroo.

Waxan bilaabay in aan tiriyo maanta (taariikhda, maalinta, bisha, sannadka) __ __ . __ __ . __ __

Waxa la ii qabtay in aan umulo (taariikhda, maalinta, bisha, sannadka) __ __ . __ __ . __ __

Waxa sida caadiga ah aan tiriyaa inta u dhexaysa saacadda: __ __ . __ __ iyo saacadda: __ __ . __ __

Saacadda 1.aad — 5 daqiiqo halkii sanduuqba.

Saacadda 2.aad — 5 daqiiqo halkii sanduuqba.

Maalmaha toddobaadka	Maalinta 1.aad	Maalinta 2.aad	Maalinta 3.aad	Maalinta 4.aad	Maalinta 5.aad	Maalinta 6.aad	Maalinta 7.aad	Maalinta 1.aad	Maalinta 2.aad	Maalinta 3.aad	Maalinta 4.aad	Maalinta 5.aad	Maalinta 6.aad	Maalinta 7.aad	Maalinta 1.aad	Maalinta 2.aad	Maalinta 3.aad	Maalinta 4.aad	Maalinta 5.aad	Maalinta 6.aad	Maalinta 7.aad	Maalinta 1.aad	Maalinta 2.aad	Maalinta 3.aad	Maalinta 4.aad	Maalinta 5.aad	Maalinta 6.aad	Maalinta 7.aad	Maalinta 1.aad	Maalinta 2.aad	Maalinta 3.aad	Maalinta 4.aad	Maalinta 5.aad	Maalinta 6.aad	Maalinta 7.aad	Maalinta 1.aad	Maalinta 2.aad	Maalinta 3.aad	Maalinta 4.aad	Maalinta 5.aad	Maalinta 6.aad	Maalinta 7.aad	Maalinta 1.aad	Maalinta 2.aad	Maalinta 3.aad	Maalinta 4.aad	Maalinta 5.aad	Maalinta 6.aad	Maalinta 7.aad
Saacadda 1.aad: START TELLING 0-5																																																	
6-10																																																	
11-15																																																	
16-20																																																	
21-25																																																	
26-30																																																	
31-35																																																	
36-40																																																	
41-45																																																	
46-50																																																	
51-55																																																	
56-60																																																	
Saacadda 2.aad: 0-5																																																	
6-10																																																	
11-15																																																	
16-20																																																	
21-25																																																	
26-30																																																	
31-35																																																	
36-40																																																	
41-45																																																	
46-50																																																	
51-55																																																	
56-60																																																	
In ka badan 2 saacadood																																																	
	Toddobaadka 28.aad							Toddobaadka 29.aad							Toddobaadka 30.aad							Toddobaadka 31.aad							Toddobaadka 32.aad							Toddobaadka 33.aad							Toddobaadka 34.aad						

Daqiiqado

F/G: Iskutallaabta godka waxa aad ku samaynaysaa qalin biiroo.

Marka foomka la buuxiyo, u dir ama u gee (cinwaanka).

Maalmaha toddobaadka

Daqiiqado

Toddobaadka	Maalmaha toddobaadka	0-5	6-10	11-15	16-20	21-25	26-30	31-35	36-40	41-45	46-50	51-55	56-60	0-5	6-10	11-15	16-20	21-25	26-30	31-35	36-40	41-45	46-50	51-55	56-60	In ka badan 2 saacadood
Toddobaadka 42.aad	Maalinta 7.aad																									
	Maalinta 6.aad																									
	Maalinta 5.aad																									
	Maalinta 4.aad																									
	Maalinta 3.aad																									
	Maalinta 2.aad																									
	Maalinta 1.aad																									
Toddobaadka 41.aad	Maalinta 7.aad																									
	Maalinta 6.aad																									
	Maalinta 5.aad																									
	Maalinta 4.aad																									
	Maalinta 3.aad																									
	Maalinta 2.aad																									
	Maalinta 1.aad																									
Toddobaadka 40.aad	Maalinta 7.aad																									
	Maalinta 6.aad																									
	Maalinta 5.aad																									
	Maalinta 4.aad																									
	Maalinta 3.aad																									
	Maalinta 2.aad																									
	Maalinta 1.aad																									
Toddobaadka 39.aad	Maalinta 7.aad																									
	Maalinta 6.aad																									
	Maalinta 5.aad																									
	Maalinta 4.aad																									
	Maalinta 3.aad																									
	Maalinta 2.aad																									
	Maalinta 1.aad																									
Toddobaadka 38.aad	Maalinta 7.aad																									
	Maalinta 6.aad																									
	Maalinta 5.aad																									
	Maalinta 4.aad																									
	Maalinta 3.aad																									
	Maalinta 2.aad																									
	Maalinta 1.aad																									
Toddobaadka 37.aad	Maalinta 7.aad																									
	Maalinta 6.aad																									
	Maalinta 5.aad																									
	Maalinta 4.aad																									
	Maalinta 3.aad																									
	Maalinta 2.aad																									
	Maalinta 1.aad																									
Toddobaadka 36.aad	Maalinta 7.aad																									
	Maalinta 6.aad																									
	Maalinta 5.aad																									
	Maalinta 4.aad																									
	Maalinta 3.aad																									
	Maalinta 2.aad																									
	Maalinta 1.aad																									
Toddobaadka 35.aad	Maalinta 7.aad																									
	Maalinta 6.aad																									
	Maalinta 5.aad																									
	Maalinta 4.aad																									
	Maalinta 3.aad																									
	Maalinta 2.aad																									
	Maalinta 1.aad																									

Saacadda 1.aad

Saacadda 2.aad

KA QAYB QAADO CILMIBAADHIS KU SAABSAN XIDHIIDHKA KA DHEXEEYA DHAQDHAQAAQA GUJO EE ILMAHA UURKA KU JIRA IYO CAAFIMAADKA ?

Waxa aanu jecelay in aan kaa codsano in aad ka qayb qaadato cilmibaadhistan adiga oo soo xeraynaya galkan marka aad umusho ka dib – haddii aad ilmo dhasho. Arrintani way ka madax bannaan tahay oo kuma xidhna in aad isticmaashay foomkaaga gujada iyo in kale.

KUMA IYO WAAYO?

Hay'adaha "Folkehelseinstituttet" (Mac-hadka caafimaadka bulshada), Xarunta cilmibaadhista daawada la xidhiidha arrimaha dhalmada ee Dhakhtarka "Rikshospitalet", Waaxda caafimaadka haweenka ee dhakhtarada "Rikshospitalet – Radium hospitalet" iyo haweenka fayo-dhowrka ayaa waxa ay wadajir u wadaan baadhitaan ku saabsan xidhiidhada ka dhexeeya dhaqdhaqaaqa gujo ee uu ilmuhu ku sameeyo caloosha hooyada iyo caafimaadkiisa mustaqbal. Waxa aynu og nahay in hoos u dhaca xoogga ah ee dhaqdhaqaaqa gujo ama dhaqdhaqaaq la,aantu ay ku xidhiidhsan tahay halis xanuun iyo dhimasho ilmaha ku iman karta, waxase aynaan wax badan ka ogayn sida arrintan iyo warbixinaha kale ee ku saabsan dhaqdhaqaaqa gujo ee ilmuhu caloosha ku sameeyo loogu isticmaali karo horumarinta caafimaadka ilmaha wakhtiga dhow iyo kan dheer labadaba.

WAAD U MADAX BANNAAN TAHAY KA QAYBQAADASHADA, ISLA MARKAANA WAXA AAD UGA QAYB QAADAN KARTAA SI YAR AMA SI XOOG AH.

Hawshan cilmibaadhista waxa u fiican in inta ugu badani ay soo xerayso galkan inta aanay ka bixin qaybta dhalmada/baxnaanada umusha ka dib. Galkani qiimo badan ayuu u leeyahay cilmibaadhista, xataa haddii aanad isticmaalin foomka gujada ama haddii aanad doonayn in aad warbixin ka bixiso naftaada. Laakiin dabcan faa'iidadu kor ayay u kacaysaa mar kasta oo wax ku darsigaagu bato. Haddii aad ka yar tahay 18 sano jir, waxa habboon in aad weydiiso waalidkaaga. Adiga ayaa dooranaya inta aad doonayso in aad ku darsato barnaamijka.

MA DOONAYSAA IN AAD AHAATO QOF AAN LA GARANAYN?

Buuxi markaa warbixinaha ku qeexan dhinaca ugu hoosaysa ee bogga, isla markaana halkan iskutallaab saar si aad noo siiso ogolaanshahaaga: ☐

INTAN WAX KA BADAN MA KU DARSAN KARTAA ?

Waxa aad bixin kartaa ogolaansho si loogu xidho warbixinta galkaaga Diiwaanka dhalmada ee caafimaadka (Medisinsk fødselsregister)adiga oo dhiibaya lambarka dhalashadaada isla markaana saxeexaya halkan:

Saxeex: ________________________ Lambarka qofka ("shanta lambar ee ugu danbeeya lambarka dhalashada").

☐☐ ☐☐ ☐☐ ☐☐☐☐☐

AMA

Haddii aad ka qayb qaadanayso barnaamijka "Baadhitaanka hooyada iyo ilmaha norwejiga" waxa aad bixin kartaa ogolaansho si warbixinta galkaaga loogu xidho baadhitaanka adiga oo dhiibaya lambarka dhalashadaada, halkana saxeexaya:

Saxeex: ________________________ Lambarka qofka ("shanta lambar ee ugu danbeeya lambarka dhalashada").

☐☐ ☐☐ ☐☐ ☐☐☐☐☐

MA LAGU KALSOON KARAA KA QAYB QAADASHADA ?

Mac-hadka caafimaadka bulshada ayaa ka masuul ah ilaalinta sirta iyo ammaanka warbixintaada. Mac-hadku waxa uu ogolaansho ka haystaa hay'adda ilaalinta warbixinta (Datatilsynet), isla markaana waxa uu talo bixin ku saabsan ilaalinta warbixinaha ku saabsan adiga iyo ilmaahaaga ka helaa guddida anshaxa cilmibaadhista (Forskningsetisk komité).
Marka ugu horraysa ee Mac-hadka caafimaaadka bulshadu uu diiwaangeliyo warbixintaada oo uu isku xidho adiga iyo warbixinta aad ogolaanshaha ka bixisay, ayuu warbixintaada ka dhigayaa mid aan la garanayn cidda ay khusayso. Taa macnaheedu waxa weeye galkaaga wuu baabi'inayaa, dhammaan warbixinta qofka lagu garan karana waa la masaxayaa inta aan loo gudbin cilmibaadhayaasha.

YAAN U DHIIBAYAA GALKA WARBIXINTA?

Dhammaan qaybaha dhalmada- iyo baxnaanada waxa ku yaalla sanduuqyo si fiican loo calaamadiyey. Waydii shaqaalaha haddii shaki kaa galo.

Ma doonayo in aan warbixin bixiyo...................... ☐

Ma doonayo in aan warbixin bixiyo.................... ☐☐
(toddobaadka 40.aad baa lagu umulaa)

HALKEE KU UMUSHAY?

Dhalmo dabeeci ah .... ☐
Qalliin hore loo qorsheeyey .... ☐
Qalliin la go'aamiyey intii fooshu socotay .... ☐
Qalliin degdeg ah .... ☐
Nooca ilmaha Wiil ☐ Gabar ☐
Miisaanka ilmuhu ku dhashay ☐☐☐☐ garaam

XAALADDA ILMAHA KA WARRAN?

Ilmuhu wuu caafimaad qabay .... ☐
Ilmaha waxa la dhigay qaybta carruurta hadda dhalatay / qaybta carruurta ee dhakhtarka ☐
Ilmihii wuu dhintay .... ☐

Da'daada .... ☐☐ sano
Miisaankaaga uurka ka hor ....☐☐☐ kg buuxa
Dhererkaaga ....☐☐☐ cm buuxa

Imisa carruur ah ayaad hore u dhashay? ☐☐

Miyaad sigaar cabtay bishii ugu dambaysay ee uurkan?
Maya ...... ☐
marmar ... ☐ ☐☐ Sigaar todobaadkii
Maalin walba .. ☐ ☐☐ Sigaar maalintii

Afkaaga hooyo ma ka duwan yahay norwejiga ?
HAA ☐ MAYAI ☐
Haddii jawaabtu HAA tahay, magacii afkaaga hooyo? ☐☐☐☐☐☐☐☐☐☐

---

folkehelseinstituttet

Rikshospitalet – Radiumhospitalet HF

Waad ku mahadsan tahay caawimada!

Oversatt ved Tolketjenesten i Oslo.
November 2005

[somali]

...Gujada ilmaha uurku ma aha farxad muujin keliya ee...

www.kickscount.no
