# Supplementary material for: Reduction of late stillbirth with the introduction of fetal movement information and guidelines – a clinical quality improvement
Source: BMC Pregnancy Childbirth. 2009 Jul 22;9:32. doi: 10.1186/1471-2393-9-32 (PMC2734741; doi:10.1186/1471-2393-9-32)
Supplement: Additional file 6 — Kicks Count. Kicks Count brochure, Urdu version. A brochure of information aiming to increase maternal awareness and vigilance to significant decreases in fetal activity, and to aid health promoting behavior. The brochure was provided as a part of the routine information given to women at the standard ultrasound assessment at 17–19 weeks in Norway as a part of the quality improvement intervention. [file 1471-2393-9-32-S6.pdf]

میرے بچے کی پیدائش کی متوقع تاریخ (تاریخ، مہینہ اور سال) ☐☐.☐☐.☐☐

میں نے آج سے گنتی شروع کی ہے(تاریخ، ماہ اورسال) ☐☐.☐☐.☐☐

میں اس وقت گنتی کرتی ہوں۔ بجے:☐☐.☐☐ بجے: ☐☐.☐☐

ضروری ہدایت: خانے میں پین سے کراس لگائیں۔

ہفتے کے دن: دن 1، دن 2، دن 3، دن 4، دن 5، دن 6، دن 7

گنتی شروع کریں

منٹ

پہلا گھنٹہ
ہر باکس 5 منٹ کا ہے

دوسرا گھنٹہ
ہر باکس 5 منٹ کا ہے

منٹ	ہفتہ 28	ہفتہ 29	ہفتہ 30	ہفتہ 31	ہفتہ 32	ہفتہ 33	ہفتہ 34
**پہلا گھنٹہ**							
0-5							
6-10							
11-15							
16-20							
21-25							
26-30							
31-35							
36-40							
41-45							
46-50							
51-55							
56-60							
**دوسرا گھنٹہ**							
0-5							
6-10							
11-15							
16-20							
21-25							
26-30							
31-35							
36-40							
41-45							
46-50							
51-55							
56-60							
2 گھنٹے سے زیادہ							

حرکت شمار کر کے خیریت معلوم کرنا؟

روزانہ حرکت محسوس کرنے کی کوشش کرنا اچھی عادت ہے اور حرکت شمار کرنے کا چارٹ ایک آسان ذریعہ ہے جس سے آپ کو اور آپ کی دائی/ڈاکٹر کو بچے کی حرکت کا جائزہ حاصل ہو جاتا ہے۔ اس سے آسانی سے پتہ چل جاتا ہے کہ آپ کے بچے کیلئے کیا نارمل ہے۔ اگرچہ حرکت سب کیلئے اہم ہے، حرکت شمار کرنے کا چارٹ حمل کے 28 ویں ہفتے کے بعد استعمال کرنا زیادہ مناسب ہے نیز تب مناسب ہے جب آپ کے پیٹ میں جڑواں بچے نہ ہوں۔ اگر آپ حرکت کا چارٹ بھر کر ولادت کے بعد دے دیں تو آپ اس اہم تحقیق میں بھی مددگار بنیں گی کہ بچے کی حرکتوں کی بنیاد پر حمل کے وہ کیس کیسے دریافت کئے جائیں جن میں اضافی مدد کی ضرورت ہے۔ اگر آپ اس تحقیق میں شامل ہونا چاہتی ہیں تو اس فائل کی پچھلی طرف درج معلومات پڑھیئے۔

حرکتوں کا شمار کیسے کیا جاتا ہے؟

جب آپ حمل کے 28 ویں ہفتے تک پہنچ جائیں تو حرکتیں شمار کرنا شروع کر دیں۔آغاز کی تاریخ اور ولادت کی متوقع تاریخ چارٹ پر لکھیں۔

10تک گنیں۔ پہلی مرتبہ حرکت محسوس ہونے کے بعد سے وقت نوٹ کرنا شروع کریں (تاکہ آپ کو پتہ چل جائے کہ بچہ جاگ رہا ہے)۔ تمام حرکتیں "ضربات " کے زمرے میں آتی ہیں البتہ آپ ہچکی کو شمار نہ کریں! بیک وقت کئی حرکتیں ہوں تو انہیں ایک "ضرب یا ایک حرکت" شمار کریں۔ اگر آپ لیٹ جائیں/ سکون سے بیٹھ جائیں اور خوب دھیان دیں تو یہ کام جلدی پورا ہو جائے گا۔ اگر بچہ سو رہا ہو تو اسے جگانے میں کوئی ہرج نہیں۔ اسے جگانے کیلئے آپ اپنے پیٹ کو تھوڑا تھپتھپائیں/دھکیلیں یا کوئی ٹھنڈا مشروب پی لیں۔

چارٹ کے خانے میں اتنے وقت پر کراس لگائیں جو 10 مرتبہ حرکت محسوس کرنے میں لگا اور دن پر بھی نشان لگائیں۔

جہاں تک ممکن ہو، روزانہ تقریبا ایک ہی وقت پر گنا کریں۔ دن یا رات کا کوئی ایسا وقت چن لیں جب آپ کیلئے وقت نکالنا آسان ہو اور آپ کو پتہ ہو کہ بچہ اس وقت حرکت کیا کرتا ہے – صبح بستر سے نکلنے سے پہلے بہتر رہے گا۔ ہر روز انہی دو گھنٹوں کے اندر گنتی شروع کیا کریں۔ چارٹ پر لکھیں کہ آپ کس وقت گنتی کرتی ہیں۔

ان باتوں پر عمل کرنے والی اکثر خواتین کو حرکتیں شمار کرنے میں 15 منٹ سے کم لگتے ہیں۔

گنتی فارم کس طرح بھرا جائے:

نیلے/کالے پین سے خانے میں کراس (کاٹا) لگائیے۔ جب آپ 10 مرتبہ حرکت شمار کر چکیں تو آپ اس خانوں والے چارٹ میں کراس لگا کر واضح کریں کہ اس میں کتنا وقت لگا۔

مثال:

مثال کے طور پر اگر آپ کو 10 مرتبہ حرکت محسوس کرنے میں 30 منٹ لگے ہوں تو آپ چارٹ کے اس خانے میں کراس لگائیں 26-30 منٹ۔

اگر آپ کو 10 مرتبہ حرکت شمار کرنے میں 1 گھنٹہ اور 5 منٹ لگے ہوں تو آپ دوسرے گھنٹے والے حصے میں 0-5 منٹ پر کراس لگائیں۔

	دن 1	دن 2	دن 3	دن 4	دن 5	دن 6	دن 7
0-5							
6-10							
11-15							
16-20							
21-25	X						
26-30		X					
31-35							
36-40							
41-45							
46-50							
51-55							
56-60							

	دن 1	دن 2	دن 3	دن 4	دن 5	دن 6	دن 7
0-5			X				
6-10							
11-15							
16-20							
21-25							
26-30							
31-35							
36-40							
41-45							
46-50							
51-55							
56-60							
2 گھنٹے سے زیادہ							

بچے کو کتنی حرکت کرنی چاہیئے اور اگر حرکت کم ہو جائے تو؟

ایک یا دو ہفتے حرکت کا چارٹ استعمال کرنے کے بعد آپ دیکھیں گی کہ ہر روز بچے کی حرکتوں میں کچھ فرق ہوتا ہے لیکن پھر بھی عمومی طور پر یہ دن ایک جیسے ہوتے ہیں۔ ایک صحتمند بچے کی کیفیت یہی ہوتی ہے خواہ حمل کے دوران آپ کو حرکتیں مختلف طور پر محسوس ہوتی رہیں۔ سب سے اہم بات یہ ہے کہ آپ کے بچے کی نارمل حرکت میں بہت زیادہ یا مستقل کمی نہیں آنی چاہیئے! حرکتوں کے چارٹ سے آپ کیلئے یہ دیکھنا آسان ہو جاتا ہے۔
اگر آپ کو اپنے بچے کے بارے میں تشویش ہے تو وجہ خواہ کچھ بھی ہو، آپ کو مشورہ اور مدد طلب کرنی چاہیئے۔ اگرآپ کو اس وجہ سے فکر ہے کہ ہفتے گزرنے کے ساتھ ساتھ آپ کے بچے کی حرکت بتدریج کم ہوتی جا رہی ہے تو اگلے معائنۂ حمل کیلئے آپ بچے کی حرکت کا چارٹ ساتھ لے آئیں۔
بعض صورتوں میں آپ کو براہ راست ہسپتال کے شعبہ پیدائش سے رابطہ کرنا چاہیئے:

- اگر کسی دن آپ کا بچہ حرکت نہ کرے تو ہرگز اگلے دن تک انتظار نہ کیجئے۔
- اگر ایک دن یا کئی دنوں کے دوران بچے کی حرکت لگاتار کم ہوتی جائے اور آپ کو "زندگی کا کم احساس ہو"۔

اگر آپ کو ٹھیک سے معلوم نہ ہو کہ "زندگی کا کم احساس " ہونے کا کیا مطلب ہے تو یہ سمجھ لیجئے کہ ایسا انتہائی کم ہوتا ہے کہ ایک صحتمند بچہ اپنےمعمول میں چست رہنے کے وقت میں دو گھنٹے کے دوران 10 مرتبہ سے کم حرکت کرے۔ اگر آپ کو محسوس ہو کہ سارا دن بچے نے بہت کم حرکت کی ہے تو آپ کو ہسپتال کے شعبہ میٹرنٹی سے رابطہ کرنا چاہیئے۔ اگر آپ کو یہ معلوم نہ ہو کہ کیا اس دن کی گنتی شروع کرنے سے پہلے بھی بچے کی حرکت بہت کم رہی ہے تو آپ کو دھیان رکھنا چاہیئے۔ اگلے 12 گھنٹوں کے اندر اندر ضرور دوبارہ حرکتیں گنیں اور اگر نتیجہ پہلے کی طرح برآمد ہو تو ہسپتال کے شعبہ میٹرنٹی سے رابطہ کریں۔

اپنے بچے سے دوستی کیجئے!

اب آپ کے حمل کو اتنا عرصہ گزر چکا ہے کہ شاید آپ کو بچے کی ضربات یا حرکات محسوس ہوتی ہوں گی۔ معائنہ حمل سے آپ کو بچے کی صحت اور خیریت کا تو پتہ چلتا رہتا ہے لیکن ماں کی حیثیت سے آپ ہی ہیں جو بچے کی پیدائش سے پہلے اس کی سب سے قریبی دوست بن سکتی ہیں۔ بچہ اپنی حرکت کے ذریعے جو کچھ بتاتا ہے، وہ اہم ہے!

ولادت کے بعد آپ کا بہت سا وقت بچے کو سنبھالنے میں گزرا کرے گا۔ ہمارا آپ کو مشورہ ہے کہ آپ حمل کے دوران روزانہ تھوڑا سا وقت اپنے بچے کی حرکتوں کے ذریعے اس سے جان پہچان حاصل کرنے میں گزاریں۔ یہاں آپ کو ایسی معلومات دی جا رہی ہیں کہ بچے کے حرکت کرنے کا کیا مطلب ہے اور یہ رہنمائی کی جا رہی ہے کہ آپ اپنے بچے کی صحت اور اطمینان کا کیسے جائزہ لے سکتی ہیں۔

یہ اچھی عادت ہے کہ اپنے بچے سے جان پہچان بنانے کیلئے کچھ وقت مخصوص رکھا جائے!

بچے کی حرکت سے اس کی خیریت کے بارے میں کیا پتہ چلتا ہے؟

بچے کو آپ سے ضرورت کی تمام چیزیں پلاسینٹا کے ذریعے ملتی ہیں۔ جب تک بچے کو درست فراہمی جاری رہے، اس کی حرکت جاری رہتی ہے۔ اگر پلاسینٹا سے بچے کی ضروریات درست طور پر پوری نہ ہوں یا وہ بیمار ہو جائے تو اسے اپنی نشوونما جاری رکھنے کیلئے اپنی طاقت بچا کر رکھنا پڑتی ہے لہذا اس کی حرکت کم ہو جاتی ہے۔ اگر آپ تمباکو نوشی کرتی ہیں تو اس سے پلاسینٹا اور بچے، دونوں پر اثر پڑے گا اور بچے کی حرکت کم ہو جائے گی۔ اگر پلاسینٹا کے فعل میں شدید خلل پڑ گیا تو نشوونما رک جائے گی اور بچے کے شدید بیماری یا نقائص میں مبتلاہونے کا خطرہ ہے۔ اس صورت میں بچہ پیدائش کے عمل کو بھی ٹھیک طرح برداشت نہیں کر پاتا۔ اگر بچے کی حرکت نارمل انداز میں جاری رہے تو ایسا بہت ہی کم ہوتا ہے۔

کبھی کبھار بالکل سکون رہتا ہے۔ جو بات اہم ہے، وہ یہ ہے کہ معمول کی حرکت بہت کم ہو جائے۔ جب آپ کو اپنے اندر زندگی کا عام ردھم محسوس ہوتا رہے تو یہ بچے کی خیریت کی علامت ہے۔ بچے کی ضربات محسوس ہونے سے آپ کو اپنے بچے سے مزید لگاؤ محسوس ہوتا ہے۔ اس لئے بچے کی حرکتوں کو گن کر اس کی خیریت معلوم کرتے رہنا اچھا ہے!

اس لئے ہم حرکات کی گنتی کرتے ہیں!

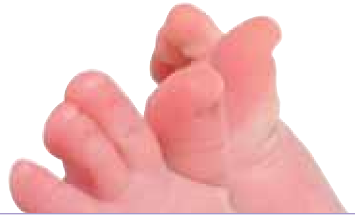

جب بچہ پیٹ میں ضربات لگائے تو اس کا کیا مطلب ہے؟ہے؟

بچہ اندر کیا کر رہا ہے؟

آپ کا بچہ حمل کا تمام عرصہ حرکت کرتا رہتا ہے۔ اس کی سب سے عام حرکت سانس لینے سے واقع ہوتی ہے جو آپ کو محسوس نہیں ہوتی۔ اس طرح پھیپھڑے پھیلتے ہیں اور بچہ پیدائش کے بعد کے وقت کیلئے مشق کرتا رہتا ہے۔ کبھی کبھار اسے ہچکی لگ جاتی ہے جو آپ کو لگاتار ہلکے ہلکے جھٹکوں کی طرح محسوس ہوتی ہے۔

بچہ آپ کے پیٹ میں چھوٹی بڑی، سبھی طرح کی حرکتیں کرتا رہتا ہے۔ ننھی سی گرفت، انگوٹھا چوسنا یا ہاتھوں پیروں کی انگلیوں کو موڑنا اور پھیلانا تو آپ کو محسوس نہیں ہوتا ہو گا۔ البتہ بچے کی بڑی جسمانی حرکات جیسے پاؤں مارنا یا دھکیلنا آپ کو حمل کے آخری حصے میں بڑی اچھی طرح محسوس ہوتی ہیں۔ بڑی جسمانی حرکات تو اکثر واضح ہوتی ہیں اور ایسی صورتوں میں واقع ہوتی ہیں جیسے اگر آپ کھڑی رہنے کے بعد لیٹنے لگیں۔ ایسے میں بچہ تھوڑا سا مڑ جاتا ہے اس لئے اسے اپنی پوزیشن تھوڑی سی بدلنی پڑتی ہے یعنی اگر آپ بچے کو تھوڑا سا دھکیلیں گی تو وہ بھی واپس دھکیلے گا۔

کبھی کبھار حرکت نہیں ہوتی . . .

آپ کا بچہ دن میں کئی مرتبہ سوتا ہے اور تب وہ بالکل پرسکون رہتا ہے۔ حمل کا عرصہ بڑھنے کے ساتھ ساتھ بچے کی نیند کے اوقات بڑھتے جاتے ہیں لیکن ولادت کے قریب آ کر بھی کم ہی ایسا ہوتا ہے کہ بچہ ایک وقت میں ایک گھنٹے سے زیادہ سوئے۔ زیادہ تر بچے شام کے وقت زیادہ چست ہوتے ہیں اور کئی بچے صبح سویرے بھی چست ہوتے ہیں۔

مختلف بچوں کی "ضربات" کی کثرت اور طاقت میں بہت فرق ہو سکتا ہے ضربات سے یہاں ہماری مراد ہر قسم کی حرکات ہیں۔ جو بچے زیادہ حرکت کرتے ہیں، وہ اکثر پیدائش کے بعد بھی زیادہ چست رہتے ہیں۔ تاہم وہ بھی اتنے ہی صحتمند ہوتے ہیں۔ لڑکوں اور لڑکیوں میں فرق نہیں پایا جاتا۔

بعض ماؤں کو دوسری ماؤں کی نسبت حرکت اتنی اچھی طرح محسوس نہیں ہوتی۔ اگر پلاسینٹا رحم کے سامنے کی طرف لگا ہوا ہو یا آپ کا جسم بھاری ہو تو آپ کو کم محسوس ہوتا ہے۔ آپ کو جب پیٹ ہلتا ہوا نظر آئے تو آپ ساتھ ساتھ حرکت محسوس کرنے کی کوشش کیا کریں۔ جب آپ لیٹی ہوں تو حرکت سب سے زیادہ محسوس ہوتی ہے، کھڑے ہوئے، چلتے ہوئے یا مصروفیت کے دوران حرکت سب سے کم محسوس ہوتی ہے۔

فوٹو : Per Oscar Skjellnan • www.peroscar.no
ڈیزائن: Marianne Bratt Ricketts • www.mariannedesign.no

کیا آپ کوئی سوال پوچھنا چاہتی ہیں؟ دیکھیے www.telltrivselen.no

[urdu]

پیٹ میں بچے کی حرکت
محض کھیل اور مزا نہیں

kicks count

www.kickscount.no

پیٹ میں بچے کی حرکت اور صحت کے باہمی تعلق کے بارے میں تحقیق میں شمولیت

اگر آپ نے ایک بچے کو جنم دیا ہے تو ہم آپ کو دعوت دیتے ہیں کہ بچے کی پیدائش کے بعد آپ یہ فائل مکمل کر کے دے دیں اور اس طرح تحقیق میں شامل ہو جائیں۔ اس سے کوئی فرق نہیں پڑتا کہ آپ نے بچے کی حرکتوں کا چارٹ استعمال کیا ہے یا نہیں۔

کون اور کیوں؟

Folkehelseinstituttet (انسٹیوٹ اف پبلک ہیلتھ) ، Rikshospitalet کے دوران حمل طبی حالات کے تحقیقی مرکز یعنی Perinatalmedisinsk forskningssenter، Kvinneklinikken Rikshospitalet-Radiumhospitalet HF یعنی ان دو ہسپتالوں سے منسلک خواتین کلینک اور Sanitetskvinnene (نارویجن عورتوں کی تنظیم برائے عوامی صحت) کے تحت یہ تحقیق کی جا رہی ہے کہ پیٹ میں بچوں کی حرکت اور ان کی آئندہ صحت کا آپس میں کیا تعلق ہے۔ ہم جانتے ہیں کہ حرکت میں شدید کمی آنے یا حرکت بند ہو جانے سے بیماری اور موت کا خطرہ ہوتا ہے لیکن ہمیں اس بارے میں زیادہ علم نہیں کہ ان معلومات اور پیٹ میں بچے کی حرکت کے بارے میں دیگر معلومات کو مستقبل قریب و بعید میں بچوں کی صحت کے فروغ کیلئے کس طرح استعمال کیا جا سکتا ہے۔

تحقیق میں شامل ہونا آپ کی اپنی مرضی پر منحصر ہے اور آپ خواہ تھوڑی مدد کریں یا زیادہ، ٹھیک ہے۔

تحقیق کیلئے یہ اہم ہے کہ شعبہ پیدائش / زچہ بچہ سے رخصت ہونے سے پہلے زیادہ سے زیادہ عورتیں یہ فائل بھر کر دیں۔ یہ فائل تحقیق کیلئے اس صورت میں بھی فائدہ مند ہے کہ آپ نے حرکتوں کا چارٹ استعمال نہ کیا ہو یا آپ اپنے بارے میں معلومات نہ دینا چاہتی ہوں۔ البتہ آپ جتنی زیادہ معلومات دیں گی، اس کی قدر و قیمت بھی اتنی ہی بڑھ جائے گی۔

اگر آپ کی عمر 18 سال سے کم ہے تو اپنے سرپرستوں سے پوچھیے۔آپ یوں طے کر سکتی ہیں کہ آپ کس حد تک حصہ لیں گی:

کیا آپ اپنا نام خفیہ رکھنا چاہتی ہیں؟

اس صورت میں آپ نیچے دیئے گئے سوالات کا جواب لکھ دیجئے اور یہاں کراس لگا کر اپنی طرف سے اجازت دے دیجئے : ☐

کیا آپ زیادہ مدد کر سکتی ہیں؟

آپ اپنا شناختی(پرسنل) نمبر یہاں درج کر کے اور دستخط کر کے اجازت دے سکتی ہیں کہ آپ کی فائل سے حاصل کردہ معلومات کو میڈیکل رجسٹر پیدائش میں شامل کر لیا جائے:

دستخط: ____________________ شناختی نمبر: ☐☐ ☐☐ ☐☐ ☐☐☐☐☐

یا

اگر آپ 'ماں اور بچے کے نارویجن سروے' میں حصہ لے رہی ہیں تو آپ اپنا پرسنل نمبر یہاں درج کر کے اور دستخط کر کے یہ اجازت دے سکتی ہیں کہ آپ کی فائل سے معلومات حاصل کر کے سروے میں شامل کر لی جائیں:

دستخط: ____________________ شناختی نمبر: ☐☐ ☐☐ ☐☐ ☐☐☐☐☐

کیا اس تحقیق میں شامل ہونا محفوظ ہے؟

انسٹیوٹ آف پبلک ہیلتھ (Folkehelseinstituttet) کی ذمہ داری ہے کہ آپ کی معلومات کو صیغۂ راز میں اور محفوظ رکھے۔ انسٹیوٹ نےڈاٹا کے حصول کے نگران ادارے (Datatilsynet) سے اجازت اور اخلاقیاتی کمیٹی برائے تحقیق (Forskningsetisk komité) سے آپ کے اورآپ کے بچے کے بارے میں معلومات محفوظ رکھنے کیلئے ہدایات حاصل کی ہیں۔ جونہی انسٹیٹوٹ آپ کی معلومات رجسٹر کرلے گا اور جہاں آپ نے معلومات آگے فراہم کرنے کی اجازت دی ہے، وہاں معلومات مہیا کر لے گا تو ان معلومات سے آپ کا نام حذف کر دیا جائے گا۔ اس کا مطلب ہے کہ محققین کو معلومات پہنچنے سے پہلے آپ کی فائل ضائع کر دی جائے گی اور آپ کی شناخت کے بارے میں تمام معلومات غائب کر دی جائیں گی۔

میں فائل کہاں دوں؟

تمام ہسپتالوں کے شعبہ پیدائش اور شعبہ زچہ بچہ میں اس مقصد کیلئے ڈبے موجود ہیں جن پر وضاحت سے لکھا ہوا ہے۔ اگر آپ کو ٹھیک سے سمجھ نہ آئے تو عملے سے پوچھ لیجئے۔

میں کوئی معلومات نہیں دینا چاہتی .... ☐

حمل کے کس ہفتے میں آپ نے بچے کو جنم دیا؟.. ☐☐
(ہفتہ 40 میں ولادت ہوتی ہے)

آپ نے بچے کو کیسے جنم دیا؟

عام ولادت(براستہ شرمگاہ)........................... ☐
بڑا آپریشن، پہلے سے طے شدہ.................... ☐
بڑا آپریشن جس کا فیصلہ دوران ولادت کیا گیا...... ☐
بڑا آپریشن جو جلدی میں کرنا پڑا................. ☐

بچے کی جنس لڑکا ☐ لڑکی ☐
پیدائش کے وقت بچے کا وزن ☐☐☐☐ گرام

بچے کی کیفیت؟

بچہ صحتمند تھا............................... .... ☐
بچے کو نومولودوں/بچوں کے شعبے میں داخل کیا گیا... ☐
بچہ فوت ہو گیا... .... ☐

آپ کی عمر...................................... ☐☐ سال
حمل سے پہلے آپکا وزن .... ☐☐☐ پورے کلو
آپ کا قد........... .... ☐☐☐ پورے سینٹی میٹر
آپ پہلے کتنے بچوں کو جنم دے چکی ہیں؟ ☐☐

کیا اس حمل کے آخری ماہ کے دوران آپ سگرٹ پیتی رہی ہیں؟
نہیں .... ☐
کبھی کبھار. .... ☐ ہفتے میں اتنے سگرٹ ☐☐
روزانہ.................. ☐ دن میں اتنے سگرٹ ☐☐

کیا آپکی مادری زبان نارویجن کے سوا کوئی اور ہے؟ ہاں ☐ نہیں ☐
اگر ہاں تو کون سی زبان؟ ☐☐☐☐☐☐☐☐☐☐

---

folkehelseinstituttet

Rikshospitalet – Radiumhospitalet HF

مدد کیلئے شکریہ!

Oversatt ved Tolketjenesten i Oslo.
November 2005

جب چارٹ مکمل ہو جائے تو اس پتے پر بھیج دیں یا پہنچا دیں۔ (.adr)

ضروری ہدایت: خانے میں پین سے کراس لگائیں۔

ہفتے کے دن

منٹ

		ہفتہ 35	ہفتہ 36	ہفتہ 37	ہفتہ 38	ہفتہ 39	ہفتہ 40	ہفتہ 41	ہفتہ 42
		دن 1 – دن 7	دن 1 – دن 7	دن 1 – دن 7	دن 1 – دن 7	دن 1 – دن 7	دن 1 – دن 7	دن 1 – دن 7	دن 1 – دن 7
پہلا گھنٹہ	0-5								
	6-10								
	11-15								
	16-20								
	21-25								
	26-30								
	31-35								
	36-40								
	41-45								
	46-50								
	51-55								
	56-60								
دوسرا گھنٹہ	0-5								
	6-10								
	11-15								
	16-20								
	21-25								
	26-30								
	31-35								
	36-40								
	41-45								
	46-50								
	51-55								
	56-60								
	2 گھنٹے سے زیادہ								
